# Vaccine-Induced Protection Against Furunculosis Involves Pre-emptive Priming of Humoral Immunity in Arctic Charr

**DOI:** 10.3389/fimmu.2019.00120

**Published:** 2019-02-04

**Authors:** Laura M. Braden, Shona K. Whyte, Alyson B. J. Brown, Carter Van Iderstine, Corinne Letendre, David Groman, Jeff Lewis, Sara L. Purcell, Tiago Hori, Mark D. Fast

**Affiliations:** ^1^Hoplite Laboratory, Department of Pathology and Microbiology, Atlantic Veterinary College, University of Prince Edward Island, Charlottetown, PE, Canada; ^2^Department of Veterinary Sciences, Universite de Montreal, Montreal, QC, Canada; ^3^Centre for Aquaculture Technologies Canada, Souris, PE, Canada

**Keywords:** furunculosis, RNAseq, Arctic charr, *Aeromonas salmonicida*, complement, vaccine, aquaculture

## Abstract

With respect to salmonid aquaculture, one of the most important bacterial pathogens due to high mortality and antibiotic usage is the causative agent of typical furunculosis, *Aeromonas salmonicida* spp. *salmonicida* (*Asal*). In Atlantic salmon, *Salmo salar*, the host response during infections with *Asal* is well-documented, with furunculosis outbreaks resulting in significant mortality in commercial settings. However, less is known about the host-pathogen interactions in the emerging aquaculture species, Arctic charr *Salvelinus alpinus*. Furthermore, there is no data on the efficacy or response of this species after vaccination with commonly administered vaccines against furunculosis. To this end, we examined the immunological response of *S. alpinus* during infection with *Asal*, with or without administration of vaccines (Forte Micro®, Forte Micro® + Renogen®, Elanco Animal Health). Artic charr (vaccinated or unvaccinated) were i.p.-injected with a virulent strain of *Asal* (10^6^ CFUs/mL) and tissues were collected pre-infection/post-vaccination, 8, and 29 days post-infection. Unvaccinated Arctic charr were susceptible to *Asal* with 72% mortalities observed after 31 days. However, there was 72–82% protection in fish vaccinated with either the single or dual-vaccine, respectively. Protection in vaccinated fish was concordant with significantly higher serum IgM concentrations, and following RNA sequencing and transcriptome assembly, differential expression analysis revealed several patterns and pathways associated with the improved survival of vaccinated fish. Most striking was the dramatically higher basal expression of complement/coagulation factors, acute phase-proteins, and iron hemostasis proteins in pre-challenged, vaccinated fish. Remarkably, following *Asal* infection, this response was abrogated and instead the transcriptome was characterized by a lack of immune-stimulation compared to that of unvaccinated fish. Furthermore, where pathways of actin assembly and FcγR-mediated phagocytosis were significantly differentially regulated in unvaccinated fish, vaccinated fish showed either the opposite regulation (ForteMicro®), or no impact at all (ForteMicro®Renogen®). The present data indicates that vaccine-induced protection against *Asal* relies on the pre-activation and immediate control of humoral immune parameters that is coincident with reduced activation of apoptotic (e.g., NF-κB) and actin-associated pathways.

## Introduction

Outbreaks of disease caused by parasitic, viral, and bacterial pathogens are a critical factor impeding sustainable growth of global finfish aquaculture. Vaccine development for viral and parasitic aquatic pathogens have lagged behind the growth of the industry due to a paucity of information regarding vaccine efficacy, host-pathogen interactions, and host biology (1). In contrast, there are many vaccines currently available in commercial applications for the control of bacterial pathogens [reviewed in (1)]. However, the basic mechanisms involved in the vaccine-associated protection against these pathogens are not well-understood.

Furunculosis is a systemic disease of salmonid and non-salmonid fishes caused by the Gram-negative bacillus *Aeromonas salmonicida* subspecies *salmonicida* (hereafter referred to as *Asal*). Originally described in 1890 (2), furunculosis has been a significant source of mortality of cultured fish worldwide (3) and is a major cause of antibiotic usage in commercial aquaculture. Furunculosis has several presentations, from chronic infections that result in pathological symptoms including lethargy, darkened skin, loss of appetite, and the development of boils or furuncles on the skin and musculature, to acute infections that are usually associated with juvenile fish and results in rapid septicemia and necrotic lesions of the epidermis (4). This latter form of the disease is accompanied by significant mortality [reviewed by (5)]. Pathological symptoms are due in part to actions of the type III secretion system (T3SS), which provides the bacteria with a mechanism to inject effector proteins into host cells, resulting in immune evasion through inhibition of intracellular killing and phagocytosis (6, 7), as well as by glycerophospholipid cholesterol acyltransferase complexed with lipopolysaccharide (GCAT/LPS) (8, 9).

There are intra- and interspecific differences in susceptibility to *Asal* among salmonids. For example, chum (*Oncorhynchus keta*), coho (*Oncorhynchus kisutch*), and chinook *(Oncorhynchus tshawytscha*) salmon exhibit variability in furunculosis susceptibility (10); brown trout (*Salmo trutta*), Atlantic salmon (*Salmo* salar), and brook trout (*Salvelinus fontinalis*) are more susceptible to *Asal* infection in comparison with other species; and, intra-specific resistance to infection exists among different populations of steelhead salmon (*Oncorhynchus mykiss*), brown trout, and Atlantic salmon (11, 12). Variable genetic host responses have been correlated with improved survival following *Asal*-infection. For instance, proteomic and transcriptomic analysis of *Asal*-infected rainbow trout spleen revealed significant induction of iron-regulating proteins (e.g., ferritin), pathogen-recognition receptors (e.g., CD209), and anti-inflammatory cytokines (e.g., IL-13/4) (13). Hepatic transcriptome analysis correlated survival in vaccinated Atlantic salmon with decreased expression of a number of transcripts involved in recruitment and motility of immune cells, including leukocyte cell-derived chemotaxin, annexins, and integrin binding proteins (14). Furthermore, resistant Atlantic salmon have significantly higher haemolytic activity pre-challenge than susceptible salmon, and survival is correlated with Th2-type responses (15). Moreover, there are several examples of higher survival correlated with specific allelic variants or genotypes in Atlantic salmon (16, 17).

*Aeromonas salmonicida* has also been isolated from Arctic charr (*Salvelinus alpinus*), and outbreaks of furunculosis are known to occur in farming operations (18). Arctic charr is a salmonid that exhibits the most northern distribution among species in this family and has evolved to tolerate extremely cold temperatures, likely due to elevated plasma electrolyte concentrations and altered epidermal characteristics (19). Due to their remarkable phenotypic and life history variation, Arctic charr have been proposed as the most variable vertebrate species (20). There is an existing commercial fishery for Arctic charr in Canada, and over the last decade, a modest effort to grow Arctic charr commercially resulted in ~10,000 metric ton production globally. Commercial strains are based on three wild populations—Nauyuk Lake and Tree River strains from Nunavut, and the Fraser River strain from Labrador—which are genetically differentiated from each other (21, 22).

For the last 20 years, incidence of furunculosis has been reduced in food fish using vaccines (23, 24); however, associated protection has been inconsistent, the nature of protection is unclear, and outbreaks of *Asal* infection persistently occur. It is thought that high individual variation of responses to vaccination in Atlantic salmon, together with high diversity of *Asal* strains, and limited knowledge of mechanisms of pathogenicity are likely contributors to limited vaccine success. Earlier work demonstrated significant but comparable efficacy of Furogen-2® (Aqua Health, USA) in protection against furunculosis in two different strains of Arctic charr (24); however, the immune response responsible for associated protection was not determined. There is no comparable report for currently administered vaccines against furunculosis. Thus, improving current vaccines or development of new vaccines are contingent on a more comprehensive understanding of the molecular mechanisms underlying these host-pathogen interactions.

Over the last several years, leveraging significant advances in genetic analysis such as high-throughput RNA sequencing has become a popular avenue for understanding pathogenicity and host responses in aquaculture. For example, there have been several studies utilizing such approaches to assess the host response during *Asal* infection (15, 25). However, to date, there are no such studies reporting whole transcriptome responses of Arctic charr to *Asal*. And further, there is no data on the efficacy of vaccines currently available for the control of *Asal* (i.e., ForteMicro®, Elanco Animal Health) in this species. To this end, we performed a comparative analysis on head kidney from vaccinated and unvaccinated Arctic charr during an experimental challenge with *Asal* using high-throughput mRNA sequencing. Our analysis demonstrates that vaccination significantly improves survival of the Fraser River strain of Arctic charr during infection through marked pre-activation of innate and adaptive humoral immune factors.

## Materials and Methods

### Fish Husbandry and Vaccination

All procedures involving the handling and treatment of fish in this study were approved by the University of Prince Edward Island Animal Care and Use Committee prior to initiation and performed under the animal use permit #13-044. Arctic Charr (*Salvelinus alpinus*) juveniles (*n* = 1,500, Fraser River strain; >10 g) were obtained from a commercial supplier (Coastal Zone Research Institute) were housed in 1200 L holding tanks at 11 ± 1.0°C in a flow-through fresh well water system. Fish were fed twice daily to satiation with a commercial feed (EWOS Transfer, St. George, New Brunswick Canada) and maintained on a 14 h light:10 h dark photoperiod. Once all fish reached appropriate size (>20 g) they were sedated with tricaine methanosulfonate (TMS-222; 100 mg/L) and individually tagged with passive integrated transponder (PIT) tags. Prior to initiation of the study, fish were randomly assigned to a treatment group and separated into triplicate tanks (300 m^3^) per group (*n* = 30 per tank): Phosphate-buffered saline (PBS)-injected fish (Sham controls), ForteMicro®-vaccinated fish (FM-vaccinates), and ForteMicro®+Renogen®-vaccinated fish (FM+R-vaccinates). Fish were sedated with TMS-222 (100 mg/L) prior to being intraperitoneally (i.p.)-injected with either PBS or vaccine.

### Bacterial Culture and Infection Challenges

U11545-99, a virulent isolate of *Aeromonas salmonicida* spp. *salmonicida* (*Asal*), recovered from Artic charr with typical symptoms of furunculosis in 1999 (Aquatic Diagnostic Services, AVC) was used in this study. A sample from frozen stock was cultured in tryptic soy broth (TSB; Bacto™, Becton, Dickinson and Company, Sparks, MD, USA) at 22°C with constant shaking at 200 rpm until the cell density reached ~1 × 10^6^ cells mL^−1^ based on optical density at 600 nm (OD_600_). The suspension was centrifuged and resuspended following washing and dilution in PBS.

A pre-vaccine challenge dose titration was performed to determine a challenge dose that would result in ~60–70% mortality. Ninety (90) unvaccinated, naive fish were separated into three experimental groups, each group consisting of duplicate tanks, holding 15 fish each. Fish were maintained as previously described. Three doses of the bacterium, 10^5^,10^3^, and 10^1^ CFU per 0.1 mL were selected. Fish were anesthetized as previously described and i.p.-injected with 0.1 mL/fish of the appropriate bacterial dose. Following recovery and return to their tank of origin fish were observed at least twice daily for ~4 weeks.

Vaccinated fish were transferred to study tanks (160 L) by treatment group (*n* = 30/tank in triplicate); treatment was randomized to tank. Fish were acclimated for 2 weeks before challenge. At 517 degree-days post-vaccination (ddpv), fish were anesthetized (TMS-222; 100 mg/L), challenged i.p. with 100 μl (10^5^ CFUs/fish) bacterial suspension or sterile PBS, allowed to recover, and returned to their tank of origin.

In both the dose determination and vaccine efficacy, fish were monitored for morbidity (inability to maintain normal dorsoventral position in the water column, lack of response to stimuli, etc.) and mortalities a minimum of twice daily. Upon detection, mortalities were promptly removed, and infection established by re-isolation of the bacterium from the head kidney on Blood Agar (BA). Plates were incubated at 13°C and monitored at least once weekly for growth; plates were monitored for 10 days.

### Sampling Design

**Figure 1:**
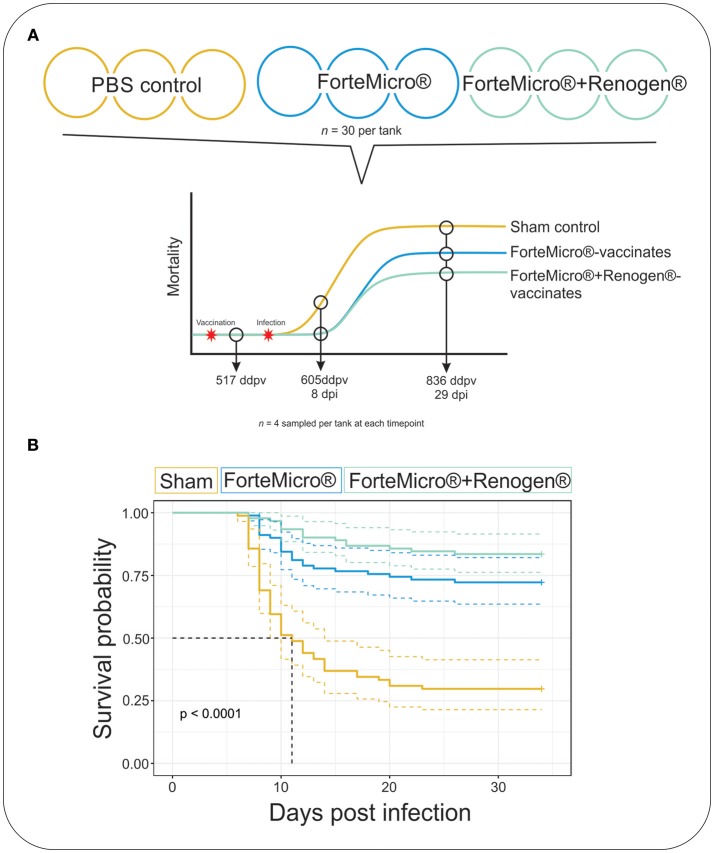
**(A)** Arctic charr were vaccinated with either 0.1 mL PBS (Sham), ForteMicro® (FM), or ForteMicro®+Renogen® (FM+R), and then i.p.-injected with *A. salmonicida* (10^6^ CFUs/mL). Each treatment was split into triplicate tanks (*n* = 30 fish per tank). Fish were sampled at 517 ddpv, 8 dpi/605 ddpv, and 29 dpi/836 ddpv. **(B)** Kaplan–Meier survival curves for Sham, FM- and FM+R-vaccinated Arctic charr over time. There was significant protection associated with vaccination of ForteMicro®, and dual administration with Renogen® further enhanced protection.

Sampling was conducted at three time points: (1) prior to infection but after vaccination (517 degree-days post-vaccination [ddpv]), (2) after mortalities were observed in all triplicate tanks of the PBS-injected Sham control (8 days post-infection [dpi]; 605 ddpv), and (3) after cessation of mortalities in all three groups (29 dpi; 836 ddpv) (Figure 1A). The experiment was terminated at 31 dpi. At each sampling time fish (*n* = 4) were opportunistically removed from each triplicate tank (*n* = 12 fish per treatment per time) and euthanized by overdose in TMS-222 (200 mg/L). Blood was sampled from the caudal vein, allowed to clot at room temperature for 30 min, centrifuged at 4,000 × *g*/4°C for 20 min, and resulting plasma was frozen at −80°C until further use. Sections of the anterior kidney, spleen, gill and proximal intestines were aseptically removed and immediately frozen on dry ice before transferring to −80°C until RNA or DNA isolation or preserved in 10% neutral buffered formalin (10% NBF).

### Enzyme-Linked Immunosorbent Assay

Antibodies directed against *Asal* were quantified using a direct ELISA. Briefly, serum samples from PBS-injected, Forte Micro®- and Forte Micro®+Renogen®-vaccinated Arctic charr underwent a serial dilution of pooled sera from each group to determine ideal concentrations for ELISA quantification. PBS-injected fish were measured at 1/128 dilution, whereas vaccinated groups were measured using a 1:5,000 dilution with 1% bovine serum albumin (BSA) in PBS-T (phosphate buffered saline with Tween 20®: 137 mM NaCl; 1 mM KH_2_PO_4_ monobasic; 8 mM Na_2_HPO_4_ dibasic; 3 mM KCl; 0.05% Tween 20 (v/v); pH 7.4) and optimized against the same strain of typical *Asal*. Flat bottomed 1 × 12 Immulon® 2HB ELISA strips (Thermo Labsystems), arranged in 96-well strip-holders were coated with *Asal* bacteria (grown to Optical Density (OD) 550 = 1.0) and coating buffer (15 mM Na_2_CO_3_, 35 mM NaHCO_3_, pH 9.6), and incubated at 4°C overnight to allow the antigen to bind. The antigen suspension was removed, and subsequently saturated with 200 μl of 3% BSA/PBS-T and incubated at room temperature for 1 h to block the remaining binding sites. Unless otherwise stated, after each step, the plate was incubated for one h at 37°C and then washed three times with PBS-T. To each well, 100 μl of diluted serum was added and allowed to incubate at 13°C for 1.5 h. The primary antibody (mouse monoclonal α-trout IgM) was diluted 1:100 with 1% BSA-PBST, and 100 μl was added to each well and allowed to incubate for 1.5 h at 37°C. The secondary antibody (1:1,000 in 1%BSA-PBST goat α-mouse IgG+M) was then added (100 μl) and incubated at 37°C for 1 h. Lastly, 100 μl of 2,2′-azino-bis(3-ethylbenzothiazoline-6-sulphonic acid) solution (ABTS)-H_2_O_2_ substrate was added per well, and incubated in the dark for 30 min at 37°C. Optical densities were measured at 405 and 490 nm on a SpectraMAX 340 plate reader (Molecular Devices).

### Nucleic Acid Isolation

#### RNA Isolation

Forty-eight samples of head kidney from three time points (0 dpi, 517 ddpv; 8 dpi, 605 ddpv; 29 dpi, 836 ddpv) consisting of three experimental conditions (Sham control, FM-vaccinates, FM+R-vaccinates; *n* = 5–7 individuals per condition) were selected for library construction, RNA-seq analysis, and RT-qPCR analysis.

RNA was extracted from frozen head kidney samples using Tri-Reagent as previously described (26), and genomic DNA contamination was eliminated after DNase treatment (Ambion® TURBO DNA-*free*™). Quality of resulting purified RNA was determined using Experion™ Automated Electrophoresis Station (BioRad) and Nanodrop 3,000 (Thermo Fisher) was used to test both purity and quantity. RNA was stored at −80°C until subsequent use.

#### DNA Isolation

DNA was isolated from 81 individual head kidney tissues (3 fish per group pre-challenge, and 4 fish per tank [triplicate tanks × 3 treatment groups × 2 post-challenge samplings]) using a Qiagen® DNeasy Blood and Tissue Kit following manufacturer's instructions with the following changes: samples (~25 mg) were homogenized for 20 min at 50 Hz (TissueLyser, Qiagen) and incubated at 56°C for 4 h. Resulting purified DNA was quantified spectrophotometrically (Nanodrop 3000) and diluted with nuclease-free water to 50 ng/ml. Integrity of gDNA was assessed on a 1% agarose gel before downstream enzymatic assays.

### Bacteriology, Histopathology, and Immunohistochemistry (IHC)

#### Bacteriology

Posterior kidneys were cultured from fish collected on designated sampling days and mortalities occurring throughout the trial by aseptically plating culture swabs on BA media at 13°C. The plates were checked at least once weekly for bacterial growth. The presence of bacterial growth, and the characteristic brown colonies on tyrosine-rich media was considered a positive criterion for typical *Asal*. Plates showing uncharacteristic growth (possible contamination) or no growth were considered negative for the presence of the bacteria.

#### Histopathology and IHC

Serial sections of paraffin-embedded formalin-fixed tissues were sectioned at 7 μm and either stained with haematoxylin and eosin (H&E) or Wright's Giemsa following standard histological techniques or probed with anti-trout IgM monoclonal antibodies using the Expose Mouse-Rabbit Specific HRP/DAB kit (Abcam) following manufacturer's instructions. Briefly, after deparaffinization and rehydration, sections were heated to 100°C in antigen retrieval buffer (pH 9) cooled to room temperature for 10 min in phosphate-buffered saline (PBS), and then washed twice in Tris-buffered saline plus 0.2% Tween 20 (TBS-T; pH 8.0) for 5 min with gentle agitation. Sections were blocked in protein blocker for 10 min before gentle rinsing with TBS-T. The sections were incubated with α-trout mIgM (1:50) in TBS-T and 1% bovine serum albumin (BSA; Sigma) overnight at 4°C in a humid chamber. After incubation, the sections were washed in TBS-T (two times for 5 min each) and incubated in a mouse-specifying reagent (Expose mouse/rabbit specific horseradish peroxidase [HRP]/3,3 = -diaminobenzidine [DAB] kit; Abcam) for 10 min, followed by a 10-min incubation in hydrogen peroxide blocker. Labeled cells were detected after a 15-min incubation in a goat anti-rabbit HRP conjugate followed by 10 min with DAB in PBS with 0.015% H_2_O_2_. All sections were counterstained in 1% Alcian blue (3 min) and Mayer's hematoxylin (diluted 1/20, 30 s), dehydrated in graded ethanol, cleared in xylene, and mounted (Permount). Sections treated with irrelevant antibodies served as negative controls, while sections known to contain IgM-labeled cells served as positive controls. After staining or immunolabeling, sections were visualized with a ZEISS AxioCam IC.

### RNA-Sequencing

#### Sequencing and *de novo* Library Construction

Total RNA was submitted for library construction and sequencing services by the McGill University and Génome Québec Innovation Center, Montréal, Canada.

RNA quality and purity were assessed using an Experion Bioanalyzer (BioRad), and only samples with a minimum RIN of 6 proceeded to library construction. Forty-eight TRUseq 100 bp pair-end stranded mRNA libraries were generated from the experimental conditions listed in Supplemental Table 1 and were sequenced in 4 lanes (12 samples per lane) on an Illumina HiSeq 2000 platform. *De novo* transcriptome assembly was performed on resulting reads following the pipeline described by Haas et al. (27) based on the Trinity assembly software suite v 2.1 (28). Briefly, reads were trimmed with Trimmomatic software (29) with a minimal Phred score of 33 and a minimal length of 32 bp. A normalized metric of reads was generated using Trinity normalization utility and surviving paired reads were assembled using the Trinity assembler (27). The final assembly quality was checked using R-correct and contigs with poor read support were removed. Trinotate v2.0.2 was used to identify putative coding transcripts and all putative transcripts were aligned against the UniProt protein database (downloaded March 2016) using the blastx program from the NCBI BLAST family as implemented in the blast+ package. Annotation was assigned to each longest putative coding transcript derived from each de Bruijn graph component (unigene) based on highest blastx score with an Expected value (E) cut-off <1e-10.

#### Mapping of Sequence Reads and Differential Expression Analysis

Read alignment to the reference transcriptome was performed using RNA-Seq by Expectation Maximization Method (RSEM) described by Li et al. (30) using the *de novo* assembled transcriptome as a reference. This method was selected as it allows for the inclusion of non-uniquely mapping reads in abundance estimates through the use of a likelihood method that accounts for read mapping uncertainty.

RSEM quantities were used as the input for the programs *edgeR* (31) and *DESeq2* (32) using scripts provided with the Trinity pipeline and a list of DE transcripts representing the mean expression values from samples for every treatment and time combination. Lists of DE transcripts generated using *DESeq2* were generally larger and were almost always contained within the *edgeR* DE lists, and for this reason we chose to use the more conservative *edgeR*-generated DE lists going forward with analysis. Differential expression was tested at a significance level of α = 0.05. Transcripts were considered differentially expressed at a *p* < 0.05, and log_2_fold-change > 2, following a false discovery rate (FDR) adjustment of 5% (0.05) as implemented by the BH algorithm in the *p.adjust* function of the R stats package.

#### Gene Ontology

Functional gene ontology was conducted using GOrilla (33) by comparing groups of differentially expressed genes to the *de novo* reference assembly as the background with an FDR-corrected *p* < 0.001. Using this open-access software, enriched GO terms are identified from ranked lists of DEGs as well as from target lists of DEGs compared to a background. Resulting enriched lists were summarized and visualized using REViGO (34) and Cytoscape.

### Conventional and Quantitative PCR

#### RNAseq Validation

To validate sequencing data, gene-specific primers were designed using Primer3 (35) and the contig sequences for nine significantly expressed transcripts, and analyzed through qPCR (Supplemental Table 2). Total RNA was extracted from samples of head kidney as described above. For every sample, 750 ng of DNase-treated total RNA was reverse transcribed using a High Capacity cDNA Reverse Transcription Kit (Thermo Fisher) following manufacturer's instructions in a reaction volume of 20 μl. qPCR was performed in triplicate reactions containing 2X Universal SYBR Green Master Mix (BioRad), 100 nm of each forward and reverse primer, and 2 μl template (diluted 1/20) in a total reaction volume of 11 μl. Cycling was performed on a CFX96 thermal cycler (BioRad) with the following thermal profile: 95°C for 30 s (1 cycle), 95°C for 15 s then 60°C for 30 s (40 cycles), followed by melt curve analyses from 65 to 95°C with fluorescence being read every 0.5 s with a ramp rate of 0.5°C to ensure amplification specificity. An equal volume of every cDNA sample was pooled and diluted 10-fold in five-steps to determine amplification efficiency and linearity for every primer pair. To confirm an absence of gDNA contamination, noRTs (no reverse transcriptase control) were performed for each gene.

Target gene expression was normalized to the two most stable of nine reference genes: *60S ribosomal protein L7* (*60S*)*, beta-2-microglobulin* (β*2m*)*, elongation factor 1-alpha* (*efI*α)*, glyceraldehyde-3-phosphate dehydrogenase* (*gapdh*)*, hypoxanthine-guanine phosphoribosyltransferase* (*hprt*)*, ribosomal protein S20* (*rps20*), *beta-actin* (ß*-actin*), *eukaryotic initiation factor 5A*(*eif5*), and *tubulin alpha chain* (*tubulin-*α). *Elongation factor 1-alpha* and *rps20* showed the highest stability, with geNorm M value and coefficient of variation of 0.993 and 0.37, respectively, using the geNorm algorithm found in qBASE^+^ software (36). Calibrated normalized relative quantities (CNRQ) were calculated with sample-specific normalization factors and gene-specific amplification efficiencies, and internal positive controls were included to calibrate run-to-run variation among plates.

Amplicons for each gene were gel purified using QIAquick Gel Extraction Kit (Qiagen) as per the manufacturer's instructions. Purified products were sent to The Center for Applied Genomics (TCAG) at the Hospital for Sick Children (Toronto, Canada) for primer verification (Supplemental Table 3).

#### Quantification of *Aeromonas salmonicida*

Specific primers for the *Asal* A449 *aopO* gene located in the low-copy-number pAsa5 plasmid (GenBank accession no. DQ386862.1) have been published previously (37). Extracted DNA from posterior kidneys of moribund and sampled fish was used to confirm *Asal* infection by conventional and real-time PCR. PCR products were synthesized using 12.5 μl GoTaq (ProMega), 2 μl of forward *aopO* primer (5′-AGCTCATCCAATGTTCGGTATT-3′), 2 μl of reverse *aopO* primer (5′-AAGTTCATCGTGCTGTTCCA-3′), 5 μl of DNA template (50 ng/μl) and 3.5 μl of nuclease-free water. The samples were run on an Eppendorf MasterCycler thermal cycler with the following thermal profile: initial heating at 95°C for 2 min, denaturation at 95°C for 1 min, annealing at 60°C for 30 s, extension at 72°C for 30 s, repeat for 34 cycles, final extension at 72°C for 5 min, and hold at 4°C. DNA from pure cultures of *Asal* was included as a positive control, whereas reactions lacking DNA template were used as negative controls. To confirm if samples were positive or negative for the *aopO* gene, PCR products were separated on a 2% agarose gel, and visualized using an ultraviolet light transilluminator (UV Transilluminator 2000, Bio Rad), with the presence of a 119-bp band considered as a positive result.

Real-time PCR was performed on a CFX96 thermal cycler (BioRad) and the CFX Manager™ software detection system (BioRad). The reaction mix contained 12.5 μl of 2X Universal SYBR Green Master Mix, 400 nM of each primer, 2 μl of template, and nuclease-free water to reach a final volume of 25 μl. The thermal cycling profile was comprised of an initial incubation of 95°C for 30 s, 35 cycles of 95°C for 15 s then 62°C for 30 s. Melt curve analysis was performed to ensure amplification specificity using a temperature gradient from 65 to 95°C with fluorescence being read every 0.5 s with a ramp rate of 0.5°C/s. Confirmed positive template were used as a positive control, while DNA extracted from fish prior to *Asal*-challenge were used as negative controls. All real-time PCR assays were performed in triplicate, and an NTC was included on every plate.

### Data Analysis

#### Survival Probabilities

The Kaplan-Meier (KM) survival curves were calculated in R using the *survival* package (R Core Developmental Team, V3.4.2).

#### Relative Proportional Survival

The relative proportion survival (RPS) was calculated for the average of the replicates for each combination of treatment factors following the formula (38):

$$\begin{array}{l} RPS=\left[1-\left(\frac{a}{b}\right)\right]x\;100\%, \end{array}$$

where a = cumulative mortalities in vaccinates

b = cumulative mortalities in non-vaccinates

ELISA data were analyzed using a two-way ANOVA followed by Tukey HSD. Correlations between mortality and antibody titer were detected using the Pearson method and performed in R (V3.4.2).

## Results

### Arctic Charr Are Susceptible to *Aeromonas salmonicida* by i.p.-injection

The challenge with *A. salmonicida* spp. *salmonicida* (*Asal*) resulted in widespread mortality of Sham controls by 8 days post-infection (dpi). At 29 dpi, mortalities were observed in all groups; however, onset of mortality was delayed in both the FM– and FM+R-vaccinated groups, with the latter displaying signs of increased protection as evidenced by a 12% increase in survival probability (Figure 1B). Dying fish presented typical signs of acute furunculosis including apathetic behavior, external hemorrhage, ascites accumulation in the peritoneal cavity, hemorrhage of the liver and body wall, hemorrhage of the stomach and pyloric caeca, enlarged spleen and liver, and swollen intestine. At the study end-date (31 dpi), there were 28.8% survivors in the Sham control group, 72.2% survivors in the FM-vaccinated group, and 83.3% survivors in the FM+R-vaccinated group (Table 1). Relative Percentage Survival (RPS) was calculated for each vaccine treatment at each time point. ForteMicro® resulted in 69.2% protection at 8 dpi and 58.3% protection at 29 dpi, while the combination of ForteMicro®+Renogen® resulted in 92.3 and 75% protection at 8 and 29 dpi, respectively (Table 1).

**Table 1:** Cumulative mortality and relative proportion survival for Arctic charr challenged with *Asal* at 8 and 29 dpi with either PBS-injection, ForteMicro®-vaccination or ForteMicro® + Renogen®-vaccination.

	**Cumulative mortality**		**Relative Proportion Survival (RPS)**	
**Treatment**	**8 dpi**	**29 dpi**	**8 dpi**	**29 dpi**
PBS	30.6 ± 17.1	71.2 ± 11.8	
Forte Micro®	8.9 ± 5.1	27.8 ± 8.4	69.2 ± 14.4%	58.3 ± 10.3%
Forte Micro®+Renogen®	2.3 ± 3.9	16.7 ± 10.6	92.3 ± 13.3%	75.0 ± 15.0%

*Values represent the average of three replicate tanks per treatment ± standard deviation of the mean*.

### Detection of *Asal*

**Figure 2:**
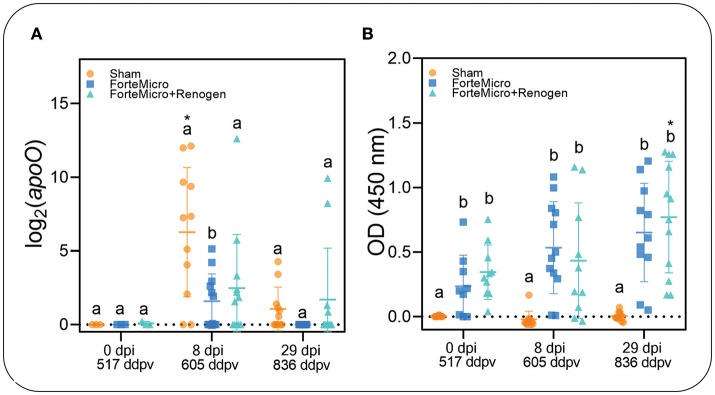
**(A)** Transcript abundance of *Asal apoO* gene was quantified over time in head kidney of vaccinated and unvaccinated Arctic charr prior to and after i.p.-injection (8 and 29 dpi). There was significantly higher expression in PBS-injected controls compared to either vaccine group at 8 dpi. At 29 dpi, expression decreased in controls to the same levels as vaccinated fish. Scatterplots are showing all samples with the mean denoted by the hashmark (*n* = 12) and the error bars indicating the standard deviation. Significance was determined by 2-way ANOVA followed by *post-hoc* Tukey's HSD (*p* < 0.05). Lower-cased letters denote significant differences among groups at each time point, while asterisk denotes differences over time within the same group. **(B)** Concentration of *Asal*-specific antibodies in charr plasma were measured by ELISA. Data shown are from 1:125 (PBS-control) and 1:5,000 (FM- and FM+R-vaccinate groups) dilution of serum samples (*n* = 9, per group). Statistical significance was determined using a two-way ANOVA followed by Tukey *post-hoc* HSD (*P* < 0.05). There was a significant difference between control and either FM- or FM+R-vaccinated charr at all-time points (*p* < 0.001).

To establish presence of *Asal* in sampled tissues, we incubated posterior kidney swabs on blood agar plates, as well as using conventional and real-time PCR to quantify the *apoO* gene as previously described (37). The kidney of all naïve Arctic charr became infected with *Asal* following i.p.-injection as determined by bacteriology, PCR, and qPCR (Figure 2A; Supplemental Table 4). Interestingly, there was incongruence in the results of the diagnostic tests, with the molecular assays appearing to be more sensitive in detecting presence of the bacteria. For example, while there was no bacterial growth detected in FM-vaccinates at 8 dpi, PCR indicated 9 of 12 fish were positive. However, quantification by qPCR revealed a significant upregulation of *apoO* in the Sham control group, but there was no difference in either FM– or FM+R-vaccinates at 8 dpi compared to 0 dpi (Figure 2). At 29 dpi, there was no bacterial growth detected in any group; however, PCR determined that all three groups were positive for *Asal*, while qPCR showed a significant downregulation of *apoO* in Sham controls. As expected, there were more positive fish in the unvaccinated Sham group at 8 dpi (83.3%), as confirmed by PCR, while the FM– and FM+R-vaccinated groups were 75 and 50% positive for *Asal* by PCR, respectively. At 29 dpi, only 50% of non-vaccinated controls were positive by PCR, while 75% of FM-vaccinates were positive. Only 16.7% of FM+R-vaccinates tested positive for *Asal* at 29 dpi.

### Humoral and Cellular Response

The results from the ELISA indicated a significant positive correlation between vaccination and the presence of *Asal*-specific antibodies (Pearson's *r* = 0.998, *p* = 0.031; Figure 2B). There were significantly higher concentrations of *Asal*-specific antibodies in both groups of vaccinated Arctic charr compared to Sham controls at all time-points in the experiment (8 dpi → 29 dpi). There was an increase in antibody concentration over time (0 dpi → 29 dpi) in vaccinated fish, however, this was only significant in the dual-vaccine group (*p* = 0.020). We did not detect a significant difference between vaccine groups.

**Figure 3:**
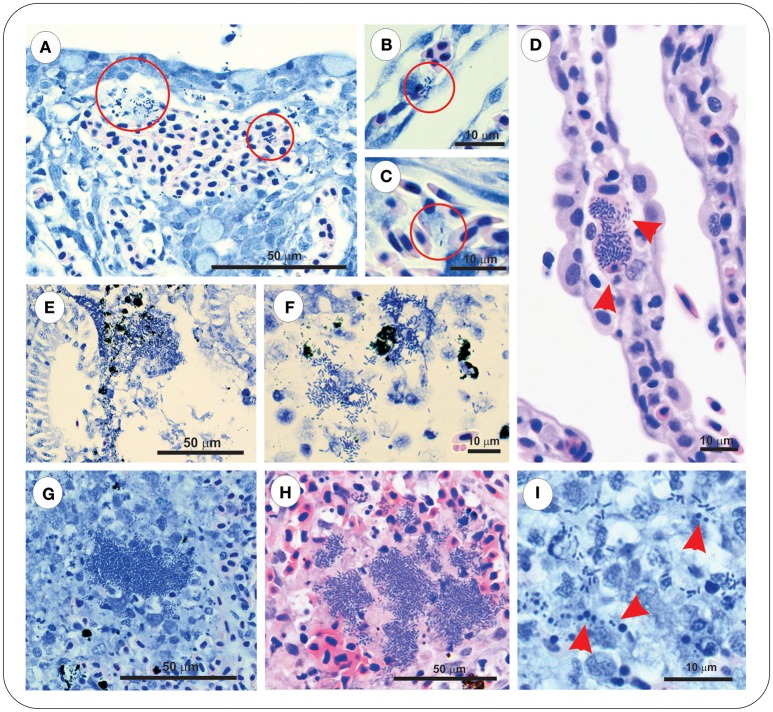
*Asal* were observed in gill, kidney and spleen of infected PBS-injected controls at 8 dpi. Gill stained with Wright's Giemsa **(A–C)** or Haematoxylin & Eosin **(D)**, showing epithelial hyperplasia and fusion of secondary lamellae with local hemorrhage and disseminated bacteria (red circles). Bacteria were also observed within vessels of intact secondary lamellae **(B)**, as well as appearing within large lymphoid cells (**C,D**, red arrowheads). Kidney of *Asal*-infected PBS-injected controls was characterized by areas of degradative necrosis and hemorrhage associated with bacterial colonies **(E,F)**. Bacterial colonies were also observed in *Asal*-infected spleen **(G,H)**, with areas of pyknotic nuclei associated with bacterial cells (**I**, red arrowheads).

Sections of gill, intestine, kidney, and spleen from FM– and FM+R-vaccinated and PBS-injected *Asal*-infected Arctic charr were stained with H&E and Wright's Giemsa. At 8 dpi, we observed evidence of pathological changes in Sham controls in all tissues examined, with bacteria present in gill, kidney, and spleen, presenting as large distinct colonies or disseminated throughout the tissue section (Figure 3). We detected several large lymphoid cells in blood vessels that appeared to harbor intracellular *Asal* bacteria (Figures 3C,D).

**Figure 4:**
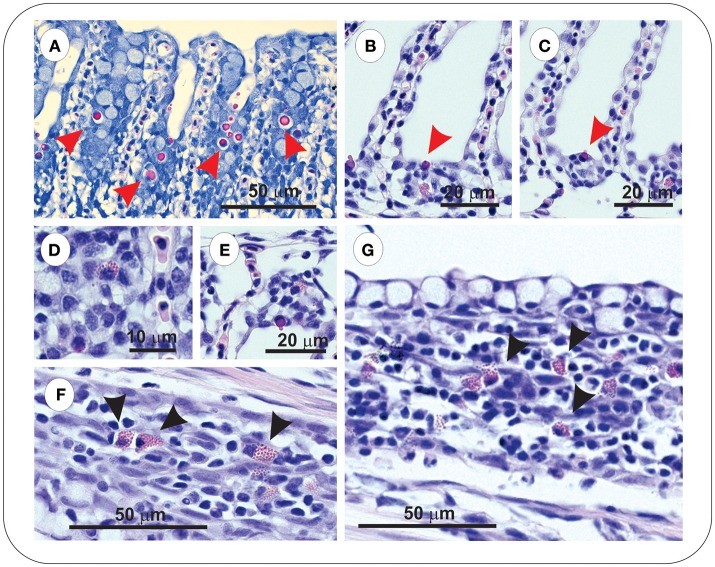
Gills of both unvaccinated and ForteMicro®-vaccinated charr were populated with sacciform cells **(A–C)**, which stained bright pink (red arrowheads). A prominent feature specific to vaccinated Arctic charr gill was eosinophilic granular cells (EGCs, black arrowheads), which were observed associated with lamellar epithelia, and interlamellar spaces **(D–G)**.

Sacciform cells were observed in the gills of both vaccinated (FM– and FM+R) and unvaccinated charr, which were present as strong eosinophilic-staining cells, often associated with a blebbing from the gill epithelium (Figures 4A–C). In *Asal*-infected fish, we observed pathological changes (e.g., filament clubbing, hypertrophy, fusion) in the gills, however, there was no apparent association between sacciform cells and gill pathology and/or the presence of *Asal* colonies. Eosinophilic granular cells (ECGs) were also observed in the gills of vaccinated *Asal*-infected charr (Figures 4D–G). These cells were more prominent in areas of lamellar thickening/fusion, in the intrabranchial lymphoid tissue (ILT) or in the gill epithelium. We were unable to quantify EGCs due to a low number of histological replicates; however, they were a conspicuous feature of FM- and FM+R-vaccinated charr and appeared to increase in number from 8 to 29 dpi, while in unvaccinated charr we failed to detect EGCs in gill tissue at any time. Eosinophilic granular cells were also observed in the intestine of *Asal*-infected charr in high numbers, both in vaccinated and unvaccinated groups (data not shown).

**Figure 5:**
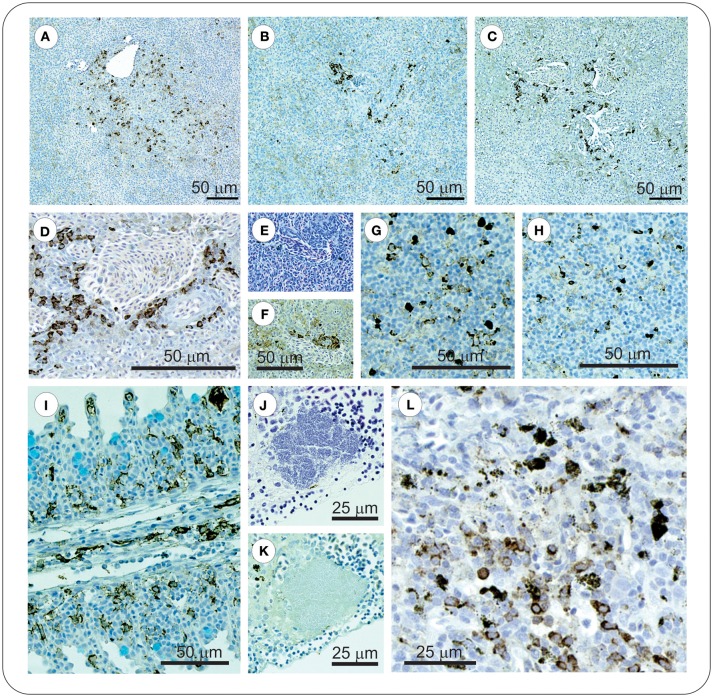
At 8 dpi positive IgM^+^ immunolabelled cells in spleen of vaccinated charr were often observed as clusters around melanomacrophage centers (MMCs) **(A–C)**, or ellipsoids **(D–F)**. In contrast, at 29 dpi, clusters of these cells were no longer evident, and instead were scattered through the spleen **(G–H)**. ForteMicro®-vaccinated charr gill was densely populated with IgM^+^ cells at 8 dpi **(I)**. In kidney of PBS-injected controls, *Asal* colonies were not associated with IgM^+^ lymphocytes **(J,K)**, however positive immunolabeled cells were observed associated with melanomacrophages **(L)**.

At 8 dpi, in the spleen of FM- and FM+R-vaccinated and Sham control groups, numerous melanomacrophages (MMs) were observed scattered throughout the red and white pulp (Figure 5). Melanomacrophages were present in arterioles and surrounding ellipsoids, and there was also evidence of apoptotic cells (pyknotic nuclei) (Figure 3I). In FM- and FM+R-vaccinated charr specifically, MMs were often associated with aggregates of IgM^+^ lymphocytes clustered around ellipsoids or inside arterioles, with higher numbers of IgM^+^ cells in the spleen of FM-vaccinates (Figures 5B,D). At 29 dpi, the spleen of FM+R-vaccinated charr was densely populated with MMs, with a smaller number of IgM^+^ clusters (Figure 5G). In contrast, at 29 dpi, MMs in the spleen of FM-vaccinates were lower in number and there were fewer IgM^+^ lymphocytes detected (Figure 5H). We did observe IgM^+^ lymphocytes in the spleen of Sham controls, however, these were very sparse and did not appear to associate with MMs or with colonies of *Asal* (Figure 5). In contrast, the kidney of Sham controls was populated with IgM^+^ cells that were associated with MMs (Figure 5).

Kidney of both Sham controls and vaccinated charr were densely populated with MMs, and in controls we observed areas of necrosis associated with bacterial colonies in the Sham group (Figures 3E,F).

Sham control intestine was characterized by numerous cells with pyknotic nuclei, mucocyte hyperplasia, hemorrhage and necrosis of the lamina propria, all of which were absent in FM- and FM+R-vaccinated fish intestine (Supplemental Figures 1A,B). Positively immunolabeled cells were observed in the lamina propria and migrating throughout intestinal mucosa (Supplemental Figure 1C).

### RNA Sequencing

#### Raw Sequencing Data and Quality Statistics

An average of 15.7 million reads per library was generated, with 73% of all reads mapping to the reference transcriptome (Supplemental Table 5). All contigs from the *de novo* assembly were analyzed using Transdecoder to identify the best putative ORF. Resulting protein sequences were annotated using the Trinotate v2.0.2 using the uniport database retrieved on March 2016.

#### Transcriptomic Response of Arctic Charr to *A. salmonicida*

RNA-seq libraries were generated from 48 samples of Arctic charr arbitrarily chosen from 9 discrete treatment conditions (Supplemental Table 1). Raw sequence data from all libraries has been deposited in the NCBI Sequence Read Archive as accession PRJNA507334.

Transcriptome *de novo* assembly yielded a total of 664,663 contigs with an average transcript length of 786 bp. These sequences represent 423,594 trinity genes, of which, ~181,321 (42.8%) were suggested as being protein-coding following Trinotate and Uniprot blastx annotation analysis (Supplemental Data File 1). Of these, 69,019 were annotated by UniProt. Abundance estimates (RSEM expected counts) for all transcripts identified in each RNA-seq library is provided in Supplemental Data File 2. To provide concordance in differential expression we first used two different pipelines (*edgeR, DESeq2*). Lists of DE transcripts generated from *DESeq2* were generally larger and fully encompassed *edgeR* DE transcript lists (Supplemental Figure 2). We decided to consider data obtained by the more conservative *edgeR* pipeline for subsequent analysis. Lists of differentially expressed transcripts identified from abundance estimates using *edgeR* are provided in Supplemental Data File 3.

**Figure 6:**
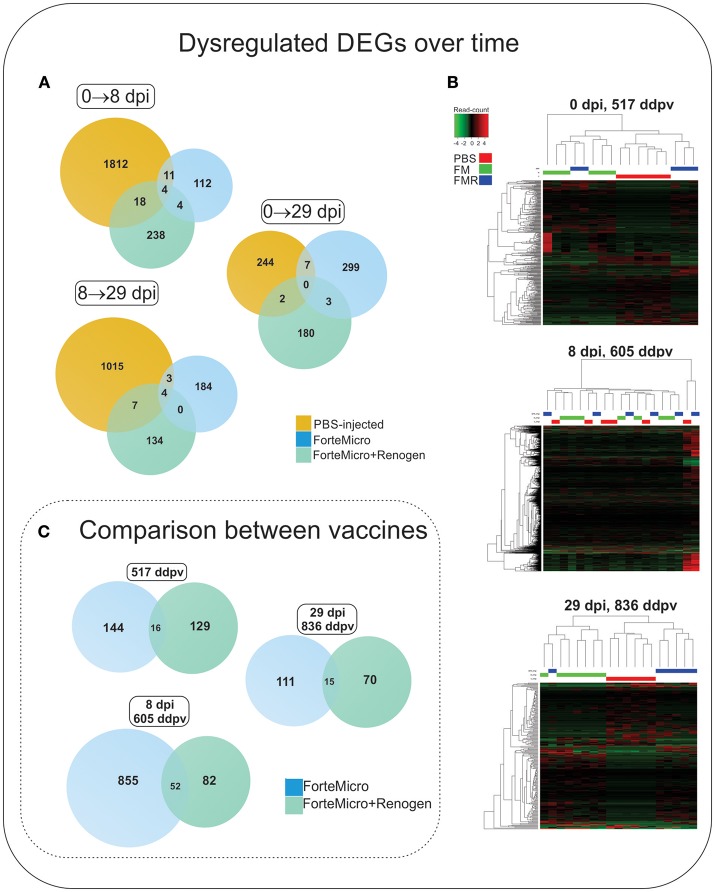
**(A)** Venn diagram of DEGs in Sham controls, ForteMicro® (FM)- and ForteMicro®+ and Renogen® (FM+R)-vaccinates over the time course of the study. **(B)** Hierarchical cluster analysis of the expression patterns of all DEGs in Sham controls, FM- and FM+R-vaccinates at each time point (*p* < 0.05). The expression values (TPM) shown are log_2_-transformed, and median-centered. Venn diagram of DEGs in FM- and FM+R-vaccinates compared to Sham controls at 517 ddpv, 8 dpi/605 ddpv, and 29 dpi/836 ddpv. **(C)** Venn diagram of DEGs in FM- and FM+R-vaccinates compared to controls prior to infection with *A. salmonicida* (517 ddpv), after infection with *Asal* (8 dpi, 605 ddpv), and in surviving fish (29 dpi, 836 ddpv).

The transcriptomic profile of both groups of vaccinated Arctic charr followed a similar pattern to that of Sham controls, characterized by low numbers of differentially regulated transcripts prior to bacterial challenge, followed by an increase in over-expressed transcripts during the bacterial infection, ending with a decrease in over-expressed transcripts in post-challenge, surviving fish. However, in contrast to Sham controls, the degree and magnitude of the response in vaccinated fish was significantly reduced. Furthermore, the transcriptomic response was observed to differ depending on the vaccine administered with a much stronger transcriptomic response in FM-vaccinates compared to FM+R-vaccinates (Figure 6; Supplemental Tables 6, 7).

#### Vaccination Induces a Primed Humoral Immune Response

To evaluate the effect of vaccination on the transcriptome, we first compared the transcriptomic profiles of vaccinated (FM or FM+R) to unvaccinated (Sham) fish prior to infection with *Asal* (517 ddpv). There were 145 and 160 differential expressed genes (DEGs) in fish vaccinated with FM+R and FM, respectively, with 19 transcripts concordantly overexpressed by both vaccinate groups (Table 2). Three of these transcripts (*40S ribosomal protein S12, ubiquitin carboxyl-terminal hydrolase 3*, and *7SK snRNA methylphosphate capping enzyme*) were expressed in opposing directions (i.e., up-regulated in one group while down-regulated in the other).

**Table 2:** Concordantly over-expressed transcripts in both vaccinated groups (ForteMicro® and ForteMicro®+Renogen®) prior to bacterial challenge, showing the log_2_-transformed fold-change compared to unvaccinated fish (FDR-corrected *p*-value < 0.05).

**Transcript**	**Forte Micro®**	**Forte Micro® + Renogen®**
Monocyte to macrophage differentiation factor 2	11.8	13.0
Serum amyloid A-1 protein	9.4	9.7
Caprin-1	9.9	8.0–9.5
Nuclear receptor coactivator 4	10.0	6.8–9.3
N-acetyltransferase 10	9.7	9.2
Neurotensin receptor type 1	8.1	8.3
Cartilage acidic protein 1	8.2	8.0
Phospholipid-transporting ATPase ID	8.1	8.0
CREB-regulated transcription coactivator 2	7.5	7.6
F-box/LRR-repeat protein 13	8.2	6.9
Alpha-adducin	6.8–7.0	6.7
Cobra venom factor	8.3	3.4
Integrin alpha-9	−6.5	−6.5
F-box only protein 18	−7.9	−7.9
Protein LBH	−8.2	−8.2
Ankyrin repeat domain-containing protein SOWAHC	−8.5	−8.5
40S ribosomal protein S12^*^	−6.9	10.6
Ubiquitin carboxyl-terminal hydrolase 3^*^	−7.3	7.9
7SK snRNA methylphosphate capping enzyme^*^	7.7	−8.6

*Multiple ranges indicate transcript isoforms*.

* *Differential expression between vaccine groups*.

**Figure 7:**
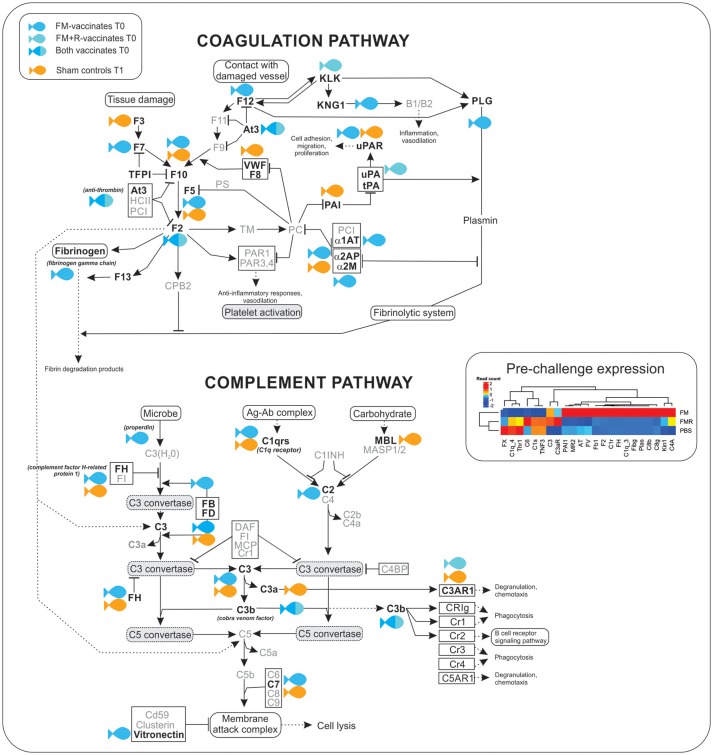
Transcripts involved in the complement and coagulation cascade were significantly up-regulated (shown in bold) in ForteMicro®- (blue symbol) and ForteMicro®+Renogen®-vaccinated (cyan symbol) fish prior to infection with *Asal* (517 dppv). In contrast, after infection there was down-regulation of genes in this pathway while in PBS-injected controls (orange symbol), these genes were up-regulated.

Compared to Sham controls, the transcriptomic response of FM-vaccinates at 517 ddpv was characterized mainly by over-expression of genes involved in the acute phase response (e.g., *alpha-2-HS-glycoprotein, serum albumin 1, ladderlectin*), complement and coagulation cascade (e.g., *complement factor H-related protein 1, complement C1r subcomponent, complement C3, alpha-2-macroglobulin;* Figure 7), and iron homeostasis (e.g., *hemopexin, serotransferrin-2, ceruloplasmin*). In contrast, FM+R-vaccinates were characterized by up-regulation of genes involved in protein synthesis (e.g., *60S ribosomal protein L13a, 40S ribosomal protein S12*), cellular transport (e.g., *choline transporter-like protein 4, phospholipid-transporting ATPase ID*) and transcription (e.g., *eukaryotic translation initiation factor 3 subunit L, nuclear receptor coactivator 4, CREB-regulated transcription coactivator 2*).

There was a high number of over-expressed transcripts associated with complement and coagulation in the FM-vaccinated charr (*n* = 13), compared to the group receiving the combined vaccination of FM+R (*n* = 3; Supplemental Table 8). Additionally, we only detected significant up-regulation of transcripts involved in the acute-phase response in FM-vaccinates prior to infection with *Asal*, such as *alpha-2-HS-glycoprotein* (*Fetuin-A*, FC = 17.3), *serum albumin 1* (FC = 13.6), *ladderlectin* (FC = 13.1), *apolipoprotein B-100* (FC = 12.5), and *apolipoprotein A-I-1* (FC = 12.4).

#### Transcriptomic Response During *Asal* Infection in Vaccinated Charr

Compared to PBS-injected controls, at 8 dpi/605 ddpv, the number of differentially expressed transcripts in FM-vaccinated fish was greater than the response in FM+R-vaccinated fish (1,163 and 145, respectively; Figure 6C, Supplemental Table 7). Of these, only 52 were concordantly over-expressed in both groups, including tissue remodeling enzymes (*matrix metalloproteinase-9*), hemostasis-associated genes (*heme-binding protein 2, hemoglobin subunit alpha*), mitochondrial enzymes (*[3-methyl-2-oxobutanoate dehydrogenase [lipoamide]] kinase*), cytoskeleton-associated transcripts (*keratin type I cytoskeletal 18, WAS/WASL-interacting protein family member 1*), and actin-binding enzymes (*gelsolin, dematin*) (Supplemental Table 9).

**Figure 8:**
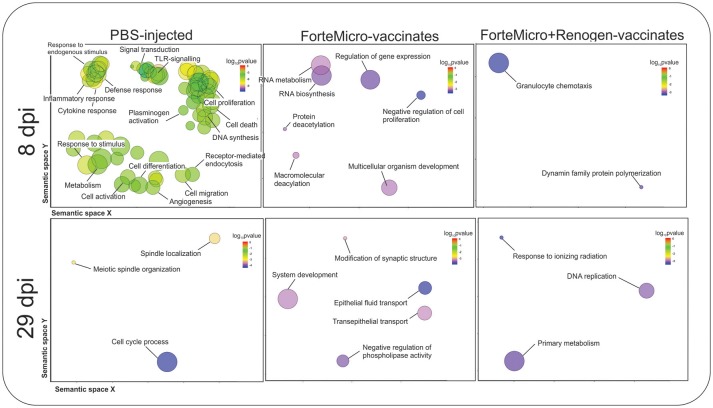
Biological processes in Sham, ForteMicro®- and ForteMicro®+Renogen®-vaccinates at 8 and 29 dpi as revealed by analysis of GO term distribution using GOrilla and REVIGO algorithms. GO terms are represented by circles and are plotted according to semantic similarities to other GO terms (adjoining circles are most closely related). Circle size is proportional to the abundance of the GO term in the list, while color indicates log10 *p*-values. Only GO terms with higher than 1% frequency are shown.

Transcripts specifically upregulated in FM-vaccinated fish were characterized by enrichment in “RNA metabolic process” (GO:0007275), “regulation of gene expression” (GO:0010468), and “multicellular organism development” (GO:0035987). In contrast, in FM+R-vaccinated fish there was enrichment of “granulocyte chemotaxis” (GO:0071621), and “dynamin family protein polymerization involved in mitochondrial fission” (GO:0003374) (Figure 8; Supplemental Data File 4).

There was no enrichment in down-regulated transcripts in FM-vaccinated Arctic charr, however, in FM+R-vaccinates there was enrichment in “respiratory electron transport chain” (GO:0022904), and “regulation of transcription of nucleolar large rRNA by RNA polymerase I” (GO:1901836).

We observed over-expression of several adaptive immunity-associated transcripts in immunized fish at 8 dpi, with a higher number of these transcripts over-expressed in FM-vaccinates. These included *BCL-6 corepressor-like protein 1* (FC = 8.54), *Ig heavy chain V region* (FC = 5.44), *Ig heavy chain V region BCL1* (FC = 8.32), and *Ig kappa chain V-I region DEE* (FC = 7.44). One transcript associated with adaptive immunity (*Ig lambda-3 chain C region*) was concordantly over-expressed as multiple isoforms in both FM- and FM+R-vaccinated fish (FC = 6.52–8.36 and FC = 3.6–8.95, respectively).

Interestingly, compared to unvaccinated controls, there was significant upregulation of a hemolytic protein, *neoverrucotoxin subunit alpha*, only in the FM-vaccinated group (FC = 8.9–10.3).

#### Transcriptomic Response in Surviving Vaccinated Fish

At 29 dpi/836 ddpv, the number of over-expressed transcripts in the FM-vaccinates decreased to a comparable level to that of FM+R-vaccinates (129 and 90, respectively; Figure 6C, Supplemental Table 7). Fifteen transcripts were concordantly over-expressed between vaccinated groups, with functions in RNA-binding (*RNA-binding protein 10*), DNA-binding (*coiled-coil and C2 domain-containing protein 1A*), DNA-repair enzymes (*tyrosyl-DNA phosphodiesterase 2*), transferases (*probable E3 ubiquitin-protein ligase HERC1*), helicases (*fanconi anemia group J protein homolog*), nuclear protein transport (*importin-7*), microtubule organization (*centrosomal protein of 170 kDa*), signal transduction (*leucine-rich repeat-containing protein 28*), and cytoskeletal organization (*bromodomain and WD repeat-containing protein 3*) (Table 3). Within these genes, three were differentially expressed between vaccine groups (*transposon TX1 uncharacterized 149 kDa protein, synergin gamma*, and *membralin)*.

**Table 3:** Transcripts that were concordantly over-expressed in both FM- and FM+R-vaccinated groups after bacterial challenge (29 dpi, 836 ddpv), showing the log_2_-transformed fold-change compared to unvaccinated fish.

**Transcript**	**Forte Micro® (FM)**	**Forte Micro® + Renogen® (FM+R)**
Coiled-coil and C2 domain-containing protein 1A	10.3	10.6
RNA-binding protein 10	10.3	9.9
Tyrosyl-DNA phosphodiesterase 2	9.2	9.2
Importin-7	8.6	9.2
Probable E3 ubiquitin-protein ligase HERC1	9.3	9.1
Neuropathy target esterase	7.9	8.0
Fanconi anemia group J protein homolog	7.5–8.8	7.8
Transposon TX1 uncharacterized 149 kDa protein^*^	−7.9	7.8
Centrosomal protein of 170 kDa	7.3	7.2
Synergin gamma^*^	−7.6	7.1
Bromodomain and WD repeat-containing protein 3	6.5	6.8
Leucine-rich repeat-containing protein 28	6.6	6.2
ATPase family AAA domain-containing protein 5	−5.3	−5.6 to (4.7)
Membralin^*^	7.4	−8.3

* Differential expression between vaccine groups

Transcripts specifically up-regulated in FM-vaccinated fish included those involved in DNA repair (e.g., *DNA cross-link repair 1A protein, fanconi anemia group J protein homolog, tyrosyl-DNA phosphodiesterase 2*), protein phosphatase/kinase activity (e.g., *protein phosphatase 1H, calmodulin*), transcriptional regulation (e.g., *coiled-coil and C2 domain-containing protein 1A, breast cancer metastasis-suppressor 1-like protein-A, polyhomeotic-like protein 1*), cell cycle regulation (e.g., *aurora kinase*), and cellular trafficking (e.g., *probable E3 ubiquitin-protein ligase HERC1, importin-7*). GO analysis detected enrichment in processes such as “negative regulation of phospholipase activity” (GO:0010519), and “epithelial fluid transport” (GO:0042045) (Figure 8; Supplemental Data File 4).

The transcriptional profile of FM+R-vaccinates also included many genes involved in the same pathways; however, in addition, we observed significant up-regulation in cellular respiration (e.g., *NADH-ubiquinone oxidoreductase 75 kDa subunit, mitochondrial, adenylosuccinate lyase, glucokinase, COX assembly mitochondrial protein 2 homolog*), adaptive immune regulation (e.g., *butyrophilin subfamily 1 member A1, nuclear factor of activated T-cells 5*), and proinflammatory regulation (e.g., *leukotriene A-4 hydrolase*).

There was significant downregulation at 29 dpi in FM-vaccinates, with enrichment in processes such as “protein activation cascade” (GO:0072376), “acute-phase response” (GO:0006953), “platelet degranulation” (GO:0002576), and “complement activation” (GO:0006956) (Supplemental Data File 4). In contrast, down-regulated genes in FM+R-vaccinates were only enriched for one biological process, “cellular senescence” (GO:0090398).

#### Infection With *Asal* Results in Massive Over-expression in Non-vaccinated Arctic Charr

There were 1,334 transcripts up- and 511 transcripts down-regulated in *Asal*-infected Sham controls at 8 dpi compared to uninfected Sham controls. Functional Gene Ontology (GO) of upregulated transcripts revealed enrichment of biological processes involved in a number of physiological functions including “cytokine-mediated signaling pathway” (GO:0019221), “regulation of cell proliferation” (GO:0042127), and “response to lipopolysaccharide” (GO:0071222) (Figure 8; Supplemental Data File 3). In contrast, enrichment in downregulated transcripts included processes such as “secretion” (GO:0046903), “exocytosis” (GO:0006887), and “regulation of reactive oxygen species metabolic process” (GO:20000377).

As the complement and coagulation cascade was highly upregulated in vaccinated fish prior to *Asal*-challenge, we were interested in comparing the expression profiles of these transcripts in unvaccinated fish over the experimental time period. Interestingly, there was high concordance in expression of many transcripts between non-vaccinated surviving charr and infected vaccinated charr (Table 4).

**Table 4:** Expression profiles of complement and coagulation-associated genes in Sham controls surviving infection (29 dpi) and FM-vaccinated fish following infection with *Asal* at 28 dpi/836 ddpv.

**Transcript**	**Surviving Controls**	**Infected ForteMicro®-vaccinates (FM)**
Antithrombin-III	–	−6.77
C3a anaphylatoxin chemotactic receptor	−3.07	
Coagulation factor X	−5.39	−7.79
Complement C1q subcomponent subunit C	4.89	5.35
Complement C1q subcomponent subunit C	−6.43	–
Complement C1q tumor necrosis factor-related protein 7	−4.64	–
Complement C1q-like protein 4	−6.17	−3.71 to (−4.57)
Complement C3	−3.49 to (−7.70)	−5.87 to (−6.67)
Complement component C7	−2.96 to (−4.84)	−4.13 to (−8.27)
Complement decay-accelerating factor	4.37–4.78	–
Complement factor D	–	−5.56
Complement factor H	−5.34 to (−7.70)	–
Fibronectin	2.46	2.74–3.57
Integrin alpha-M	–	7.61
Mannose-binding protein C	−3.18 to (−6.09)	−4.60 to (5.80)
Plasminogen activator inhibitor 1	−3.37 to (−6.34)	−3.76 to (−7.07)
Thrombospondin-1	−3.62 to (−5.98)	−4.71 to (−6.52)

*Values indicate the log_2_transformed fold change. Multiple values indicate isoforms of the same transcript*.

Compared to uninfected controls, the transcriptomic response decreased in surviving unvaccinated charr at 29 dpi, with 123 up-regulated transcripts and 130 down-regulated transcripts. Gene ontology analysis of up-regulated transcripts indicated enrichment in biological processes involved in cell-cycle process and proliferation, including “cell cycle phase transition” (GO:0044770), “mitotic cell cycle process” (GO:1903047), and “meiotic spindle organization” (GO:0000212) (Supplemental Data File 4). Enrichment of down-regulated transcripts resulted in one biological process of “negative regulation of erythrocyte differentiation” (GO:0045647) (Supplemental Data File 4).

#### Comparison Between ForteMicro®- and ForteMicro®+Renogen®-Vaccinated Charr

We then compared the transcriptomic response between the two vaccine-groups in attempts to identify differences in the host response that might account for the increased protection in the FM+R-vaccinates.

Compared to Sham controls prior to bacterial challenge (517 ddpv), there were 125 over-expressed transcripts, with 47 up-regulated in FM-vaccinated charr compared to 78 up-regulated in the FM+R-vaccinated group. Gene Ontology analysis of up-regulated transcripts in FM-vaccinates revealed enrichment in biological processes such as “acute phase response” (GO:0006953), “regulation of proteolysis” (GO:0030162), and “platelet degranulation” (GO:0002576). In contrast, FM+R-vaccinates showed no enrichment of GO terms. Enrichment of downregulated transcripts at 517 ddpv in FM-vaccinates revealed enrichment in processes including “positive regulation of lamellipodium organization” (GO:1902743), and “cellular response to biotic stimulus” (GO:0071216). In FM+R-vaccinates there was one category enriched in downregulated transcripts, “negative regulation of chromatin binding” (GO:0035562).

During the proliferative growth stage of *Asal* infection (8 dpi, 605 ddpv), 108 transcripts were over-expressed, with 62 up-regulated in FM-vaccinated fish, compared to 46 up-regulated transcripts in the FM+R-vaccinated fish. Functional GO of upregulated transcripts revealed FM-vaccinates had enrichment in “defense response” (GO:0006952), “positive regulation of establishment of T cell polarity” (GO:1903905), and “inflammatory response” (GO:0006954). In contrast, FM+R-vaccinates had enrichment of “androgen receptor signaling pathway” (GO:0030521), “positive regulation of actin nucleation” (GO0051127) (Supplemental Data File 4). In contrast, GO enrichment of downregulated transcripts of FM-vaccinated fish included processes of “chemotaxis” (GO:0006935), “regulation of signaling” (GO:0023051), and “leukocyte migration” (GO:0050900). In FM+R-vaccinates, there was much fewer enriched categories in downregulated transcripts, including “positive regulation of phagocytosis” (GO:0050766), “positive regulation of endocytosis” (GO:0045807), and “PERK-mediated unfolded protein response” (GO:0036499).

The response in surviving vaccinated fish (29 dpi, 836 ddpv) reduced to 85 over-expressed transcripts comprising 39 up-regulated in FM-vaccinates, and 46 up-regulated transcripts in FM+R-vaccinates. In FM-vaccinated fish, there was enrichment in upregulated transcripts for processes like “histone H3-K9 modification” (GO:0006413:000061647), “regulation of catabolic process” (GO:0009894), and “peptidyl-lysine monomethylation” (GO:0018026). There was no enrichment of GO terms FM+R-vaccinates in upregulated transcripts, and there was no enrichment in downregulated transcripts for either FM- or FM+R-vaccine group.

Comparison between overexpressed transcripts during the experimental time course showed that there was a similar profile between 517 ddpv and 8 dpi (~86% similarity). There were 16 transcripts specific to the pre-challenge FM-vaccinated group with significant activation in several humoral immune effectors (Table 5). In contrast, the profile of overexpressed transcripts in surviving vaccinates was discrete.

**Table 5:** Expression profiles, pre-challenge with *Asal*, of 16 distinct transcripts in ForteMicro®-vaccinates compared to ForteMicro®+Renogen®-vaccinates.

**Transcript**	**log_**2**_FC**	**Putative function**
Cytochrome b-245 heavy chain	9.89	Respiratory burst
Serotransferrin-2	11.75	Iron sequestration
Fibrinogen gamma chain	13.13	Coagulation cascade
ATP-dependent 6-phosphofructokinase, muscle type	17.75	Glycolysis
Alpha-2-HS-glycoprotein	17.39	Acute phase response
Ladderlectin	17.00	Acute phase response
Apolipoprotein B-100	15.86	Acute phase response
UPF0762 protein C6orf58 homolog	14.97	Unknown
Apolipoprotein A-I-1	14.81	Acute phase response
Type-4 ice-structuring protein	14.73	Anti-freeze protein
KxDL motif-containing protein 1	14.68	Vesicle transport
Complement C3	13.82	Complement cascade
Factor XIIa inhibitor	14.64	Coagulation cascade
Serum albumin 1	14.62	Acute phase response
Kininogen-1	14.46	Coagulation cascade
Fatty acid-binding protein 10-A, liver basic	13.86	Intracellular lipid binding

#### RNAseq Validation and Exploratory Gene Expression by qPCR

Nine genes of interest (*B-cell linker protein, mx2, il6rb, c7, ladd, fibronectin, fibrinogen gamma chain, hsp90b*) were chosen for validation with RT-qPCR on the RNA-seq samples, as well as on an expanded number of samples to increase the power of testing. Some of these genes were chosen to cross validate the DE results from RNA-sequencing, and others were of immunological importance. Fold-change comparisons between both methods revealed a significant positive correlation (Pearson's *r* = 0.60 *p* = 0.024) (Supplemental Data File 5).

We then correlated the expression of these immune associated genes with the expression of *apoO* from *Asal*. There was significant negative correlation in both *blnk* and *finc*, indicating that presence of *Asal* negatively affected expression of these genes (Supplemental Table 10).

**Figure 9:**
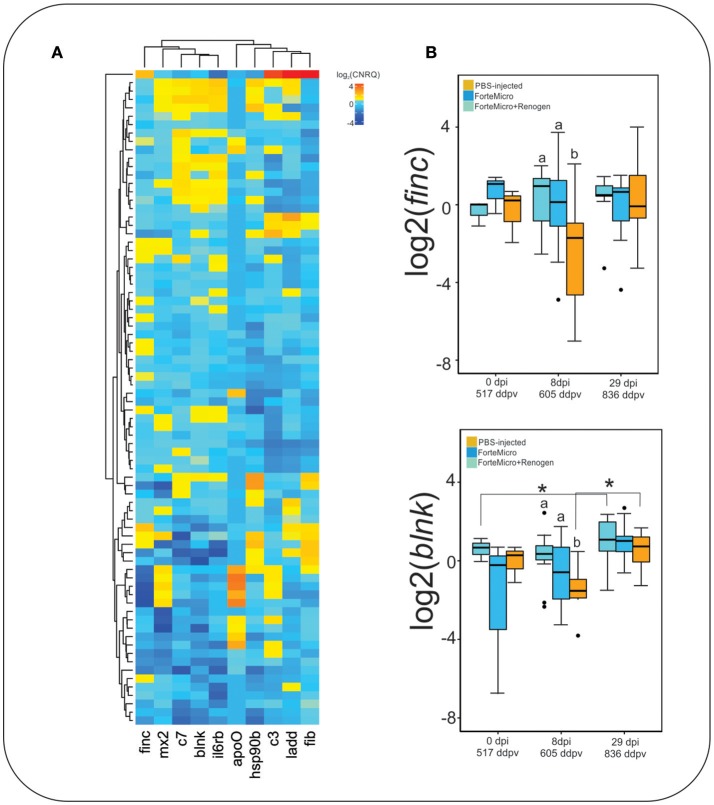
**(A)** Hierarchical clustering of expression profiles for all gene-sample combinations with qPCR. Values represent average log_2_-transformed relative quantities for all replicates of each treatment. **(B)** Expression profile of *fibronectin* and *b-cell linker protein*. Data represents the median log2CNRQ and 95% confidence intervals. Significant differences were detected with a two-way ANOVA followed by *post-hoc* Tukey HSD (*p* < 0.05). Differences within each time are denoted by lower-cased letters, while differences over time within groups are denoted by asterisk (^*^).

Acute phase response genes *ladderlectin, complement component 3*, and *complement component 7* were expressed higher in FM-vaccinates than in PBS-injected controls as identified by RNA-seq (Figure 9A). Genes involved in immunological processes such as B-cell differentiation and cytokine signaling were also confirmed to be differentially regulated through vaccination. ForteMicro®-immunized fish had a significantly higher expression of *finc* and *blnk* compared to Sham controls after infection with *Asal* (8 dpi, 605 ddpv; Figure 9B), with expression increasing in the Sham and FM+R group by the final sampling time. Other genes examined using RT-qPCR, as mentioned above, had concordant expression with the RNA-seq data, but were not all significant over time and treatment comparisons (Supplemental Figure 3).

## Discussion

Transcriptomic analyses are an informative approach to provide insight on mechanisms of host responses during infection, or to provide information on vaccine efficacy. In the present study, we used Illumina sequencing to quantify the response of Arctic charr *S. alpinus* during infection with *Aeromonas salmonicida* subsp. *salmonicida* (*Asal*) with or without administration of vaccines commonly used in salmonid aquaculture: ForteMicro® or combination of ForteMicro® + Renogen®. ForteMicro® (Elanco Animal Health) is a vaccine that contains formalin-inactivated cultures of *Aeromonas salmonicida, Vibrio anguillarum* serotypes I & II, *Vibrio ordalii* and *Vibrio salmonicida* serotypes I & II in liquid emulsion with an oil-based adjuvant. In contrast, Renogen® (Elanco Animal Health) is a live culture of a non-virulent *Arthrobacter* spp. that shares common antigenic determinants with *Renibacterium salmoninarum* and is indicated to protect salmonids from bacterial kidney disease (BKD; https://www.drugs.com/vet/renogen-can.html). However, in commercial settings, Renogen® is only administered in conjunction with anti-furunculosis vaccines such as ForteMicro®, and only in situations where BKD is a risk (39).

Both vaccine regimes resulted in significant protection to Arctic charr from *Asal*-associated mortality; however, the dual-vaccination resulted in significant improvement on the administration of ForteMicro® alone. Protection was positively correlated with elevated levels of *Asal-*specific antibodies in both vaccine groups, similar to previous reports for Atlantic salmon (40) and rainbow trout (41). Notably, significant levels of *Asal*-specific antibodies in both vaccine groups prior to bacterial challenge (over 50X > sham) indicated protective immunity was in part due to circulating antibodies. It should be stated that although dual-vaccination resulted in enhanced protection above single administration against furunculosis, this approach may come with extra cost and the current study did not examine other non-target effects of this approach.

There were patterns of differential expression between unvaccinated and vaccinated Arctic charr, as well as between the two vaccinated groups, that provides information about the effects of vaccination on the host immune response, as well as the molecular pathways involved in protection. The most remarkable pattern in this study was the significant over-expression of innate humoral molecules in Arctic charr immunized with ForteMicro®. Notably, there was significant upregulation of key regulatory components (e.g., *complement factor H, vitronectin, anti-thrombin*), as well as the genes necessary for induction of complement and coagulation (e.g., *C1q, C3, C3b, fibrinogen gamma chain, cobra venom factor*). Additionally, many genes associated with metal homeostasis (e.g., *ceruloplasmin, serotransferrin-2, hemopexin*), and the acute phase response (e.g., *fetuin-A, inter-alpha-trypsin inhibitor heavy chain H2, serum albumin 1, apolipoprotein B-100*) were significantly upregulated in this group prior to infection with *Asal*.

Surprisingly, despite elevated cumulative survival, we did not observe the same degree of activation of the complement cascade in pre-challenge fish vaccinated with the combination of ForteMicro® and Renogen® vaccines. Instead the transcriptomic response was characterized by upregulation of a single acute phase protein (*serum amyloid A-1 protein*), several cellular effector markers (e.g., *eosinophil peroxidase*), and only two complement molecules (*cobra venom factor, complement factor D*). The expression profile also included transcription factors (e.g., *eukaryotic translation initiation factor 3 subunit L, nuclear receptor coactivator 4, transcription factor E2F2*), transporters (e.g., *choline transporter-like protein 4, intermediate conductance calcium-activated potassium channel protein 4*), activators of signaling cascades (e.g., *WD repeat domain-containing protein 83*), and actin binding pathways (e.g., *alpha-adducin, Rho-related GTP-binding protein RhoE*). Interestingly, there was upregulation of a hemolytic toxin (*stonustoxin subunit alpha*) specific to pre-challenge fish vaccinated with ForteMicro® and Renogen®. Transcripts with high homology to venom factors have also been detected in the head kidney transcriptome of Atlantic salmon infected with *Asal* (42), and are known ancestral forms of perforin-like genes with pore-forming abilities and immune recognition domains (43).

After challenge with *Asal*, the transcriptome of Sham controls was characterized by a substantive and apparent unrestrained increase in expression. This observation is in agreement with reports for Atlantic salmon (44), rainbow trout (13), and turbot (45). Namely, there was exaggerated expression profiles of genes involved in innate immunity such as acute phase, inflammation, antigen presentation, cell differentiation, complement and coagulation, and wound repair. Additionally, there was an abundance of overexpressed transcripts associated with fatty-acid synthesis, hemoglobin synthesis, cell-cycle pathways and protein folding responses. Others have suggested that a strong immune response is critical for Atlantic salmon surviving *Asal* infections (15); however, this present work suggests that for the Fraser River strain of Arctic charr, a strong immune response after infection is not desirable. Overall, the present data demonstrates significant differential regulation of immune parameters in susceptible non-vaccinated charr that is otherwise absent in vaccinated fish shortly after bacterial infection, and furthermore, that protection is associated with elevated basal levels of these same parameters pre-challenge.

### *Asal* Interferes With Proinflammatory Pathways in Unvaccinated Charr

Bacterial pathogens establish within their host by circumventing immune responses and/or dysregulating apoptotic pathways (46). Type-3 secretion system (T3SS)-harboring pathogens such as *Asal* achieve this by injecting host cells with a number of virulence factors that act to interfere with critical signal transduction pathways (i.e., NFκB signaling) (47). In unvaccinated charr, we detected over-expression in several regulators of NFκB activity, including the inhibitor of κB (IκB) *B-cell lymphoma 3 protein* (*bcl-3*) which is thought to limit transcription of pro-inflammatory mediators and regulate cellular effectors (48). Significant up-regulation of several *bcl-3* isoforms was a striking transcriptomic feature of non-vaccinated charr. Thus, along with concomitant upregulation of *nf*κ*b inhibitor* α and *nuclear factor interleukin-3-regulated protein, Asal*-induced differential regulation of NFκB signaling appeared to be occurring in the susceptible PBS-injected group. Further to this, *Asal* AopP is known to induce apoptosis in affected cells (46, 49), resulting in the formation of subcutaneous wounds (i.e., furuncles) in host tissue and septicaemia (5), as is found with its closest ortholog, YopJ (46). We observed the formation of furuncles in unvaccinated charr at 8 dpi that was concomitant with over-expression of several transcripts involved in apoptosis (e.g., *tumor necrosis factor receptor superfamily member 11B, apoptosis-enhancing nuclease, BCL2/adenovirus E1B 19 kDa protein-interacting protein 3, DNA damage-inducible transcript 4 protein*). In contrast, over-expression of apoptotic-related or NFκB-associated transcripts was not evident in vaccinated charr during *Asal* infection.

Despite their important role in anti-microbial responses (50), previous work demonstrated that activation of toll-like receptors (TLRs) does not appear to be a feature of the host response against *Asal* (51). The present study reveals a similar finding, as we only detected *tlr2, tlr5*, and *tlr13* over-expression in non-vaccinated fish, while *tlr7* over-expression was observed in vaccinated fish. However, there was some interesting patterns in expression of these TLRs. For example, significant over-expression of *tlr5* was observed in non-vaccinated Arctic charr head kidney during the proliferative growth of *Asal*. TLR5 binds bacterial flagellin and activates production of inflammatory mediators in MyD88-dependent pathways (50). The activation of TLR5 during *Asal* infection is unexpected, as the bacterium is non-motile. However, the T3SS is proposed to have evolved from the flagellar apparatus (52), and thus a common antigenic determinant present in the injection apparatus may be responsible for activation of TLR5. Indeed, the transcriptomic profile of non-vaccinated charr included pronounced up-regulation of genes involved in TLR5-MyD88 pathways such as interleukins (*il3, il6, il8*, and *il10*) and chemokines (*ccl2*, and *ccl19*). Earlier work demonstrated a relationship between expression profiles of *tlr5* and resistance to *Asal* in Atlantic salmon (15). Here, only susceptible non-vaccinated Arctic charr overexpressed *tlr5* during the proliferative phase of *Asal*-infection; however, it is possible that due to the nature of our sampling design (i.e., after 8 dpi), we may have missed immediate activation of TLRs (or other immune regulators) in vaccinated fish (or unvaccinated fish), as expression of several TLRs was observed in head kidney of another salmonid species in the first 72 h post-infection with *Asal* (53). Thus, subsequent studies including more sampling events earlier during infection (i.e., 3 dpi) are necessary to capture the dynamics of immune activation during bacterial incubation. Others have suggested that during incubation, the T3SS may act to promote immunoregulatory pathways characteristic of tolerogenic dendritic cells (tDCs) and regulatory T cells (T_reg_) (54). Our data supports this hypothesis, as a prominent feature of non-vaccinated charr was over-expression of *il10*, a cytokine produced by tDCs which is responsible for potentiating an immunosuppressive T_reg_ environment (55). Orthologs to the *Asal* T3SS effector AcrV in *Yersinia* promote tDC differentiation, with resulting pathogenesis reliant upon functional IL10 (56). Further to this, Fast et al. (7) showed that T3SS deletion mutants significantly reduced production of *il10* in Atlantic salmon head kidney leukocytes. We failed to detect over-expression of *il10* in either vaccinated group, suggesting that the observed protection may be related to inhibition of *Asal*-induced immunosuppression. The mechanism for this inhibition is not fully understood, however, a novel feature of FM-vaccinated Arctic charr was the over-expression of *tlr7*, an endosomal TLR that binds to small synthetic compounds and ssRNA (57, 58), and of all the TLRs overexpressed in this study, is the only one known to activate IFN-α production by B cells and DCs (59, 60). The ability to activate DCs and thus elicit Th1 and CD8^+^ T cell responses has been exploited to enhance the efficacy of vaccination (61, 62), and this may explain the failure of *Asal* to promote a tolerogenic immune response. Significant upregulation of *tlr7* might imply that there was a vaccine-induced activation of DCs occurring, either by potentiating antigen stimulation or increased antigen uptake by DCs as suggested elsewhere (62). Further to this, significant upregulation of pore-forming perforin-like toxins (e.g., *stonustoxin subunit alpha, neoverrucotoxin subunit alpha*) in both groups of vaccinated fish supports an activation of cytotoxic activity (e.g., by CD8^+^ T cells), which would be critical for killing host cells infected with immune-evading *Asal*. Thus, the present transcriptomic data suggests that vaccination may act first through a substantive induction of antibody-mediated complement activity targeting extracellular bacteria, and secondly through cytotoxic-mediated intracellular targeting of infected self-cells. However, subsequent studies investigating the relative contributions of DCs and cytotoxic cellular effectors in *Asal-*mediated killing are required to fully understand this mechanism.

### Vaccination Primes Humoral Immunity in Arctic Charr

Activation of the coagulation cascade plays a critical role in fighting infections by entrapment and prevention of systemic dissemination of bacteria (63) and is highly conserved among vertebrates as a general immune defense mechanism against bacterial pathogens (64, 65). Our results suggest that ForteMicro® primes this response, thus preparing the fish for rapid production of proteins involved in fibrin-production. During systemic inflammation, activation of the coagulation cascade is accompanied by activation of complement (66). However, detrimental uncontrolled and simultaneous activation of complement and coagulation systems can occur during severe infections, reinforcing the necessity of tight regulation. This is achieved by inhibitors (e.g., plasminogen activating inhibitor-1, α-2-antiplasmin) that act to avoid excessive localized and systemic plasmin generation (67). In the present study, we observed significant upregulation of both activators (e.g., *cobra venom factor*) and inhibitors (e.g., α*-2-antiplasmin*) prior to bacterial infection in fish vaccinated with ForteMicro®, while there was a delay in this response in unvaccinated fish. In addition to the over-expression of coagulation components, there was also significant induction of fibrinolytic enzymes in ForteMicro®-vaccinates prior to bacterial infection. Interestingly, components of the fibrinolytic cascade were highly over-expressed in unvaccinated Arctic charr after infection with *Asal*, suggesting that uncontrolled fibrinolysis may play a role in pathogenesis, while a protective mechanism of ForteMicro®-vaccination appears to be the capacity for immediate coagulation. Earlier work indicated that a major cause of death in acute furunculosis in Atlantic salmon is circulatory failure caused by bacterial-associated coagulation (68). In agreement with Salte et al. (69), who demonstrated that prior administration of exogenous anti-thrombin and α-2-macroglobulin improve survival of Atlantic salmon with acute furunculosis, the present data indicates that priming this response also contributes to improved survival in Arctic charr.

The acute phase response was also activated in pre-challenge Arctic charr vaccinated with ForteMicro® (e.g., *serum albumin 1, C-reactive protein, fetuin-A, haptoglobin, inter-*α*-trypsin inhibitor, apolipoprotein A1, apolipoprotein B100)*, which may have helped suppress systemic inflammation. For example, fetuin-A is protective against lethal septicaemia and end toxemia in mice by directly inhibiting production of inflammatory high mobility group proteins (70), and the presence of apolipoprotein B100 was shown to inhibit growth of *Staphylococcus aurous* in mice (71). Furthermore, high-density lipoprotein apolipoprotein A1 is an inhibitor of cytokine production and may play a role as an anti-inflammatory mediator [reviewed in (72)]. We observed a negative correlation between expression of high mobility group proteins (*TOX high mobility group box family member 2* and *high mobility group protein B3*) and *fetuin-A* in non-vaccinated *Asal*-infected charr. Moreover, there was significant over-expression of serum lipoproteins *apob100* and *apoa1* (<5,000-fold) in the transcriptome of pre-challenge ForteMicro®-vaccinates. Thus, the present data suggests an effect of ForteMicro® may include suppressing mediators of lethal, systemic inflammation. Interestingly, this pattern was not present in ForteMicro®+Renogen®-vaccinates, suggesting that either the addition of the live *Arthobacter* spp. or the different formulation of the vaccine somehow abrogated this response.

Activation of metal homeostasis mechanisms was also a prominent feature in the transcriptome of ForteMicro®-vaccinated Arctic charr prior to *Asal* challenge. Controlling availability of essential transitional metals such as copper, zinc, iron and manganese to bacterial pathogens (i.e., nutritional immunity) is a critical component of successful immune responses across vertebrates, and is accomplished by transferrin's, lactoferrins, ferritins and hem proteins (73–76). An increase in transferrin and hemopexin is highly correlative with anti-microbial activity and protection against plague in mice immediately after attenuated *Yersinia pasties* vaccine administration (77), with protection involving biological activities of host iron and heme-binding proteins. Acquisition of iron by *Asal* includes siderophore-dependent and -independent mechanisms and interference of these pathways severely compromises virulence (78). In the present study, we observed evidence of nutritional immunity in both vaccinated and non-vaccinated Arctic charr. However, the pattern of expression was markedly different among the groups, with significant over-expression in pre-challenge vaccinated fish, but in unvaccinated fish, expression was only detected after infection with *Asal*. Thus, a consequence of ForteMicro® appears to include early over-expression of genes involved in nutritional immunity.

### Disruption of Host Actin Pathways in Vaccinated Fish

The bacterium is thought to enter the fish host at multiple sites, including the skin, gill, and intestine (79). The typical incubation period of *Asal* is 3–4 days, where the bacterium rapidly disseminates in kidneys, followed by the spleen, liver, and muscles (80). Virulence during this time is attributed to the T3SS, which functionally impairs cytoskeletal functions and neutralizes immune defenses [reviewed by (81)]. The T3SS is composed of Ext, Apo, Ati2, AopP, AopO, Apo (54). Of these, Ext, AopP, and Ati2 have shown functional homology with analogs of *Salmonella* and *Yersinia* spp. (81).

One important virulence mechanism of *Asal* is the effect on host cell cytoskeletal integrity as evidenced by the T3SS effector protein Ext ATPase-activating domain that acts on small monomeric Gases of the Rho family (Rho, Race, and Cdc42) and the ADP ribosylating domain that depolymerizes actin (82), causes rounding of host cells (83). Furthermore, Ati2 is involved in detachment of actin binding proteins from the plasma membrane leading to the destabilization of the host cell and its cytolysis (5). Resistance to *Asal* pathology has been associated with downregulation of cytoskeleton-associated genes such as profiling, coiling, and actin-binding proteins in Atlantic salmon (14). In a similar fashion the present data suggests that a consequence of vaccination with ForteMicro® alone or in combination with Renogen® appears to include down-regulation of Rho-associated Gases prior to and after infection with *Asal*. The Rho family of Gases is critical for successful phagocytosis in macrophages by functioning to reorganize filamentous actin, assist in NF-dB and MAPK kinase transcriptional pathways, and facilitate respiratory burst (84). Thus, T3SS-associated inhibition of actin reorganization and effector cell phagocytosis permits bacterial escapement of antibody-mediated complement. We hypothesize that by giving the host an early boost, vaccination provides the immune system time to respond effectively so that downstream toxic effects of T3SS are minimized or prevented. This same mechanism has been suggested in experimental vaccinations of channel catfish *Ictalarus punctuates* against *Flavobacterium columnare* (85). In order to demonstrate this in *Asal*-infections, subsequent studies should aim to investigate the effect of vaccination on the specific virulence factors and their impacts on host cell actin pathways.

## Conclusions

The present data suggests that the protective effect of vaccination with ForteMicro® alone or in combination with Renogen® in the Fraser River strain of Arctic charr involves immunological priming of innate humoral components that are able to combat infection immediately upon bacterial challenge. We propose that this priming of humoral innate (complement, coagulation, and metal homeostasis effectors) and adaptive (*Asal*-specific antibodies) molecules allows for a rapid aggressive response during bacterial invasion, followed by swift regulation of these same genes to help circumvent immunopathology. This two-pronged approach of enhancing extracellular binding and killing along with reduced intracellular uptake and survival resulted in significant protection from host mortality that was specific to *Asal*, but which would likely offer some level of protection against other pathogens. Future studies should investigate the contribution of individual components and particular adjuvant preparations to the host-pathways activated. It is important to note that host immune responses are likely to differ depending on the target tissue (86), and thus interpretation of the present data should consider this potential limitation. Furthermore, in order to initiate disease our infection model included an i.p.-injection of *Asal*, which differs from the natural route of infection (e.g., penetration of damaged epithelium), and which may induce a more immediate and non-specific response due to accumulation of cellular effectors at the injection site (87). Therefore, to help fully elucidate mechanisms responsible for protection against *Asal*, examination of other routes of exposure (88), differing environmental conditions (87), and potential mucosal immune induction (i.e., systemic induction of IgT) are necessary.

## Author Contributions

LB performed histological and immunohistological assays, transcriptomic data analysis and statistical analysis, and wrote the manuscript. MF and SW contributed to the conception and design of the study, performed the vaccinations, bacterial challenges, fish sampling, and contributed to writing of the manuscript. TH performed bioinformatic analysis on the RNAseq dataset. SP prepared bacterial cultures and vaccinations, helped sample fish and performed ELISA, and contributed to writing of the manuscript. AB performed qPCR assays and contributed to writing of the manuscript. All authors contributed to manuscript revision, read and approved the submitted version.

### Conflict of Interest Statement

MF reports grants from Elanco Animal Health Canada Inc., personal fees from Elanco Animal Health Canada Inc., outside the submitted work. The remaining authors declare that the research was conducted in the absence of any commercial or financial relationships that could be construed as a potential conflict of interest.
