# Supplementary material for: Vaccine-Induced Protection Against Furunculosis Involves Pre-emptive Priming of Humoral Immunity in Arctic Charr
Source: Front Immunol. 2019 Feb 4;10:120. doi: 10.3389/fimmu.2019.00120 (PMC6369366; doi:10.3389/fimmu.2019.00120)
Supplement: Supplementary file 1 [file Table_1.docx]

**Supplemental Table 1**. Samples of Arctic charr head kidney submitted for RNA-sequencing.

| **Time Point** | **Treatment** | **Number of fish** |
| --- | --- | --- |
| 0 dpi, 517 ddpv | PBS | 6 |
|  | ForteMicro | 6 |
|  | ForteMicro+Renogen | 5 |
| 8 dpi, 605 ddpv | PBS | 4 |
|  | ForteMicro | 4 |
|  | ForteMicro+Renogen | 4 |
| 29 dpi, 836 ddpv | PBS | 7 |
|  | ForteMicro | 7 |
|  | ForteMicro+Renogen | 5 |
