# Supplementary material for: Vaccine-Induced Protection Against Furunculosis Involves Pre-emptive Priming of Humoral Immunity in Arctic Charr
Source: Front Immunol. 2019 Feb 4;10:120. doi: 10.3389/fimmu.2019.00120 (PMC6369366; doi:10.3389/fimmu.2019.00120)
Supplement: Supplementary file 2 [file Table_2.docx]

| Gene Target | Name | Reference | Accession # | Forward (5'-3') | Reverse (5'-3') | Amplicon size (bp) | Efficiency  (%) |
| --- | --- | --- | --- | --- | --- | --- | --- |
| **Acute Phase Response** |  | | | | | |  |
| *interleukin-6 receptor subunit beta* | *IL-6RB* | Braden *et al.*, 2018 |  | TGGACGATGGCTTTTGGAGT | TTTCTTGCCCCAGCTCAACA | 122 | 93.9 |
| *complement component C3* | *CC3* | Braden *et al.*, 2018 |  | GGGTGGGGGTGAAATGGTAG | ACTTGCTGGAGAGTTGTCGG | 157 | 99.0 |
| *complement component C7* | *CC7* | Braden *et al.*, 2018 |  | ACGCCAAGGACGTAGTCAAG | GAGAGAGGTATCCTGGGGCA | 168 | 96.7 |
| *fibrinogen beta chain* | *FIBB* | Braden *et al.*, 2018 |  | TCGGGAATATCGCCTTCGAC | TGACTGATGCGGTCGTTACC | 82 | 105.7 |
| **Intracellular Response** |  | | | | | |  |
| *interferon-induced GTP-binding protein Mx2* | *Mx2* | Braden *et al.*, 2018 |  | CCATCCCTAAACTGGCGGAG | TACAGCCGTCCTTTTGGGTC | 163 | 91.4 |
| *heat shock protein HSP 90-beta* | *HSP90B* | Braden *et al.*, 2018 |  | TGGTGTGTTGTGACGCTGAT | AGGGTAATGCCACACGAACA | 88 | 100.0 |
| *B-cell linker protein* | *BLNK* | Braden *et al.*, 2018 |  | GTGCCTGTCTGAAGAGGACC | ACCAGTCTCGGAAACACGAC | 126 | 99.0 |
| *H-2 class II histocompatibility antigen, A-B alpha chain* | *H2AB* | Braden *et al.*, 2018 |  | AACAAAGGAGAGGGAGTGGTG | ACCCCCTGATTACCTACAGC | 92 | 85.6 |
| *BOLA class I histocompatibility antigen, alpha chain BL3-7* | *BOLA* | Braden *et al.*, 2018 |  | ACAGTCCCTCCCTCGATGTC | AACCTGTAGCGTGGCAAGTC | 70 | 88.9 |
| *interleukin-13 receptor subunit alpha-2* | *IL-13R* | Braden *et al.*, 2018 |  | GGAGGTGTGCGTTTCCTCAT | GGCAGGGATGGTGTGAACTA | 81 | 92.3 |
| **Reference Genes** |  | | | | | |  |
| *beta-actin* | *ß-actin* | Ahi *et al.*, 2013 | JR540730 | GAAGATCAAGATCATCGCCC | CAGACTCGTCGTACTCCTGCT | 122 | 94.1 |
| *beta-2-microglobulin* | *B2M* | Ahi *et al.*, 2013 | JR540731 | CGAACAGGGATGGCAGTT | TAGGTCTTCAGATTCTTCAGGTGG | 105 | 97.0 |
| *elongation factor 1-alpha* | *EF1α* | Ahi *et al.*, 2013 | JR540732 | GAAGATCGGCTATAACCCTGC | ACCTTCCATCCCTTGAACC | 111 | 99.1 |
| *glyceraldehyde-3-phosphate dehydrogenase isoform 1* | *GAPDH* | Ahi *et al.*, 2013 | JR540733 | GGTCTGATGAGCACCGTTC | GCAGGGATGATGTTCTGGC | 110 | 99.8 |
| *hypoxanthine-guanine phosphoribosyltransferase* | *HPRT* | Ahi *et al.*, 2013 | JR540734 | TTCTCAAACAGTACAACCCAAAAA | TCCTATGAAGTCTGGTGTGTAGC | 95 | 100.1 |
| *eukaryotic initiation factor 5A isoform 1* | *eIF5* | Ahi *et al.*, 2013. | JR540735 | GGCTTCGTGGTGCTGAAG | CCATGTGGACCTTAGCGTG | 91 | 99.3 |
| *60S ribosomal protein L7* | *60S* | Ahi *et al.*, 2013 | JR540736 | CATCAGGATCAGGGGTATCAA | AGCCTTGTTCAGTTTGACGAA | 109 | 97.6 |
| *40S ribosomal protein S9* | *40S* | Ahi *et al.*, 2013 | JR540737 | GAGGTGTGGAGGGTGAAGTT | CTGAGCAGGGCGTTACCTT | 113 | 97.7 |
| *ribosomal protein S20* | *RPS20* | Ahi *et al.*, 2013 | JR540738 | AGCCGCAACGTCAAGTCT | CGCAGAGTCTTTGTGGGC | 110 | 99.9 |
| *tubulin alpha chain* | *TUB* | Ahi *et al.*, 2013 | JR540739 | GTCACTACACCATTGGCAAAGA | GCTGTGGAAGATGAGGAATCC | 104 | 100.1 |
