# Supplementary material for: Vaccine-Induced Protection Against Furunculosis Involves Pre-emptive Priming of Humoral Immunity in Arctic Charr
Source: Front Immunol. 2019 Feb 4;10:120. doi: 10.3389/fimmu.2019.00120 (PMC6369366; doi:10.3389/fimmu.2019.00120)
Supplement: Supplementary file 3 [file Table_3.docx]

**Supplemental Table 3.** Sequence identity of all new qPCR assays.

| **Transcript** | **Query Cover (%)** | **E-value** | **ID (%)** | **Accession #** | **NCBI Name** |
| --- | --- | --- | --- | --- | --- |
| *blnk* | 100% | 4.00E-35 | 99% | XM_024007819.1 | *Salvelinus alpinus* B-cell linker protein (LOC111978048), transcript variant X3, mRNA |
| *mx2* | 100 | 9.00E-47 | 99 | XM_023993826.1 | *Salvelinus alpinus* interferon-induced GTP-binding protein Mx2 (LOC111968289), transcript variant X2, mRNA |
| *il10* | 100 | 5.00E-22 | 98 | XM_024140800.1 | *Salvelinus alpinus* interleukin-10-like (LOC112073524), mRNA |
| *il6rb* | 100 | 1.00E-16 | 96% | XM_014174891.1 | *Salmo salar* interleukin-6 receptor subunit beta-like (LOC106587036), transcript variant X4, mRNA |
| *h2ab* | 100 | 1.00E-10 | 95 | EF450432.1 | *Salvelinus alpinus* isolate SW3B MHC class II antigen (Saal-DAA) gene, Saal-DAA*1202 allele, exon 2 and partial cds |
| *c3* | 97 | 1.00E-36 | 97 | XM_014186867.1 | *Salmo salar* complement C3 (LOC106595495), mRNA |
| *ladd* | 100 | 7.00E-20 | 98 | XM_023966409.2 | *Salvelinus alpinus* ladderlectin (LOC111949332), mRNA |
| *dla* | 89 | 1.00E-11 | 96 | EU478851.1 | *Salvelinus fontinalis* MHC class II antigen beta chain (Safo-DAB) mRNA, Safo-DAB*0101 allele, complete cds |
| *c7* | 100 | 2.00E-43 | 98 | XM_024002090.1 | *Salvelinus alpinus* complement component C7 (LOC111974371), mRNA |
| *fibb* | 94 | 1.00E-09 | 97 | XM_024143195.1 | *Salvelinus alpinus* fibrinogen beta chain (fgb), partial mRNA |
| *il13r* | 100 | 6.00E-07 | 97 | NM_001246337.1 | Oncorhynchus mykiss IL-13 receptor-alpha-1-a precursor (il13ra1a), mRNA |
| *bola* | 75 | 2.00E-03 | 93 | HM181991.1 | Salmo salar MHC class I antigen (Sasa-UGA) mRNA, complete cds |
| *finc* | 96 | 1.00E-04 | 94 | XM_014152358.1 | Salmo salar fibronectin (LOC100380696), mRNA |
