# Supplementary material for: Vaccine-Induced Protection Against Furunculosis Involves Pre-emptive Priming of Humoral Immunity in Arctic Charr
Source: Front Immunol. 2019 Feb 4;10:120. doi: 10.3389/fimmu.2019.00120 (PMC6369366; doi:10.3389/fimmu.2019.00120)
Supplement: Supplementary file 4 [file Table_4.docx]

**Supplemental Table 4.** The presence of *Asal* was quantified by the presence of positive bacterial colonies on blood-agar plates (BacT), as well as by conventional PCR and real-time quantitative PCR targeting the *apoO* gene.

|  | **517 ddpv** | | | **8 dpi, 605 ddpv** | | | **29 dpi, 836 ddpv** | | |
| --- | --- | --- | --- | --- | --- | --- | --- | --- | --- |
| **Treatment** | **BacT** | **PCR** | **qPCR*** | **BacT** | **PCR** | **qPCR*** | **BacT** | **PCR** | **qPCR*** |
| PBS-injected | 0/10 | 0/3 | 0^a^ | 9/12 | 10/12 | 6.27±4.39^a^ | 0/12 | 6/12 | 1.06±1.48^a^ |
| FM-vaccinates | 0/10 | 0/3 | 0.07±0.13^a^ | 0/12 | 9/12 | 1.59±1.85^b^ | 0/12 | 9/12 | 0^a^ |
| FM+R-vaccinates | 0/10 | 0/3 | 0^a^ | 3/12 | 6/12 | 2.47±3.64^b^ | 0/12 | 2/12 | 1.85±3.62^a^ |

*Average log2-transformed fold-change ± standard deviation

^a-b^Significant differences among groups at each time denoted by lower-cased letters
