# Supplementary material for: Vaccine-Induced Protection Against Furunculosis Involves Pre-emptive Priming of Humoral Immunity in Arctic Charr
Source: Front Immunol. 2019 Feb 4;10:120. doi: 10.3389/fimmu.2019.00120 (PMC6369366; doi:10.3389/fimmu.2019.00120)
Supplement: Supplementary file 5 [file Table_5.docx]

**Supplemental Table 5** Summary of *de novo* assembly results of Illumina sequence data from Arctic charr head kidney using Trinity.

| **Total filtered reads** | 733,092,810 (15.727 million pairs per sample) |
| --- | --- |
| **Contigs** | 664,663 |
| **Large contigs (>1000bp)** | 146,800 |
| **N50 (bp)** | 1486 |
| **Average contig length** | 786.68 bp |
| **Reads mapped in final reference** | 73% |
