# Supplementary material for: Vaccine-Induced Protection Against Furunculosis Involves Pre-emptive Priming of Humoral Immunity in Arctic Charr
Source: Front Immunol. 2019 Feb 4;10:120. doi: 10.3389/fimmu.2019.00120 (PMC6369366; doi:10.3389/fimmu.2019.00120)
Supplement: Supplementary file 6 [file Table_6.docx]

**Supplemental Table 6.** Differentially expressed genes within Sham controls, FM-vaccinates or FM+R vaccinates over the experiment. Values indicate annotated contigs passing cut-off values of fold-change ≥ 2, FDR-corrected *p-*value < 0.05. Total number of contigs including non-annotated are presented in parentheses.

| **Group** | **Comparison** | **Up-regulated** | **Down-regulated** | **Total (non-annotated)** |
| --- | --- | --- | --- | --- |
| **Sham** | 0 vs 8 dpi | 1334 | 511 | 1845 (2260) |
|  | 8 vs 29 dpi | 397 | 632 | 1029 (1243) |
|  | 0 vs 29 dpi | 123 | 130 | 253 (298) |
| **FM-vaccinated** | 0 vs 8 dpi | 91 | 40 | 131 (151) |
|  | 8 vs 29 dpi | 58 | 133 | 191 (233) |
|  | 0 vs 29 dpi | 76 | 233 | 309 (358) |
| **FM+R-vaccinated** | 0 vs 8 dpi | 175 | 89 | 264 (307) |
|  | 8 vs 29 dpi | 50 | 95 | 145 (165) |
|  | 0 vs 29 dpi | 79 | 106 | 185 (211) |
