# Supplementary material for: Vaccine-Induced Protection Against Furunculosis Involves Pre-emptive Priming of Humoral Immunity in Arctic Charr
Source: Front Immunol. 2019 Feb 4;10:120. doi: 10.3389/fimmu.2019.00120 (PMC6369366; doi:10.3389/fimmu.2019.00120)
Supplement: Supplementary file 7 [file Table_7.docx]

**Supplemental Table 7.** Differentially expressed genes in FM-vaccinates or FM+R vaccinates compared to PBS-injected controls over the experimental period, and FM-vaccinates compared to FM+R vaccinated over time. Values indicate annotated contigs passing cut-off values of fold-change ≥ 2, FDR-corrected *p-*value < 0.05. Total number of contigs including non-annotated are presented in parentheses.

| **Time** | **Comparison** | **Up-regulated** | **Down-regulated** | **Total (non-annotated)** |
| --- | --- | --- | --- | --- |
| **0 dpi/400 ddpv** | **FM vs. PBS** | 99 | 61 | 160 (182) |
|  | **FM+R vs. PBS** | 79 | 66 | 145 (166) |
|  | **FM vs. FM+R** | 78 | 47 | 125 (148) |
| **8 dpi/468 ddpv** | **FM vs. PBS** | 325 | 628 | 953 (1162) |
|  | **FM+R vs. PBS** | 69 | 61 | 130 (145) |
|  | **FM vs. FM+R** | 46 | 62 | 108 (130) |
| **29 dpi/799 ddpv** | **FM vs. PBS** | 54 | 46 | 100 (129) |
|  | **FM+R vs. PBS** | 49 | 33 | 82 (90) |
|  | **FM vs. FM+R** | 46 | 39 | 85 (105) |
