# Supplementary material for: Vaccine-Induced Protection Against Furunculosis Involves Pre-emptive Priming of Humoral Immunity in Arctic Charr
Source: Front Immunol. 2019 Feb 4;10:120. doi: 10.3389/fimmu.2019.00120 (PMC6369366; doi:10.3389/fimmu.2019.00120)
Supplement: Supplementary file 8 [file Table_8.docx]

**Supplemental Table 8.** Up-regulated transcripts involved in complement/coagulation in vaccinated groups compared to non-vaccinated controls prior to bacterial challenge (517 ddpv). Multiple ranges indicate transcript isoforms.

| **ForteMicro®-vaccinates (FM)** | | **ForteMicro® + Renogen®-vaccinates (FM+R)** | |
| --- | --- | --- | --- |
| **Transcript** | **log_2_FC** | **Transcript** | **log_2_FC** |
| *Complement factor H-related protein 1* | 15.5-15.9 | *Complement factor D* | 7.9 |
| *Alpha-2-macroglobulin* | 14.5 | *Prothrombin* | 7.7 |
| *Complement C1r subcomponent* | 7.4-14.3 | *Kallikrein-1E2* | 7.4 |
| *Inter-alpha-trypsin inhibitor heavy chain H2* | 14.2 |  |  |
| *Alpha-1-antitrypsin homolog* | 13.9 |  |  |
| *Complement C1q-like protein 3* | 13.7 |  |  |
| *Kininogen-1* | 13.6 |  |  |
| *Complement C3* | 9.4-13.0 |  |  |
| *Vitronectin* | 11.4 |  |  |
| *Collagen alpha-1(VIII) chain* | 11.3 |  |  |
| *Fibrinogen gamma chain* | 11.3 |  |  |
| *Complement C1q-like protein 2* | 10.0 |  |  |
| *Factor XIIa inhibitor* | 7.9 |  |  |
