# Supplementary material for: Vaccine-Induced Protection Against Furunculosis Involves Pre-emptive Priming of Humoral Immunity in Arctic Charr
Source: Front Immunol. 2019 Feb 4;10:120. doi: 10.3389/fimmu.2019.00120 (PMC6369366; doi:10.3389/fimmu.2019.00120)
Supplement: Supplementary file 9 [file Table_9.docx]

**Supplemental Table 9.** Transcripts concordantly over-expressed in both FM- and FM+R-vaccinated charr after bacterial challenge (8 dpi, 605 ddpv), showing the log2-transformed fold-change(s) compared to unvaccinated fish. Multiple ranges indicate transcript isoforms.

| **Transcript** | **Forte Micro® (FM)** | **Forte Micro® + Renogen® (FM+R)** |
| --- | --- | --- |
| *Matrix metalloproteinase-9* | 14.8 | 12.9 |
| *[3-methyl-2-oxobutanoate dehydrogenase [lipoamide]] kinase, mitochondrial* | 10.9 | 10.6 |
| *WAS/WASL-interacting protein family member 1* | 9.5 | 10.0 |
| *Heme-binding protein 2* | 10.4 | 9.6 |
| *Gamma-glutamyltransferase 5** | -7.4-9.0 | 9.5 |
| *Dolichyl-diphosphooligosaccharide--protein glycosyltransferase subunit STT3B* | 9.8 | 9.4 |
| *Keratin, type I cytoskeletal 18** | -8.6-10.2 | 9.3 |
| *Gelsolin* | 10.0 | 9.1 |
| *COP9 signalosome complex subunit 5* | 9.1 | 9.1 |
| *Riboflavin transporter 2* | 8.8 | 8.9 |
| *Ig lambda-3 chain C region* | 6.5-8.4 | 3.6-8.9 |
| *Max dimerization protein 4* | 5.9 | 8.9 |
| *ATP-dependent RNA helicase DDX3Y** | 8.2 | -11.3-8.9 |
| *Multimerin-2* | 8.9 | 8.6 |
| *GTP-binding nuclear protein Ran* | 8.3 | 8.6 |
| *Rho GDP-dissociation inhibitor 1* | 10.2 | 8.5 |
| *Cyclin-dependent kinase 8* | 8.7 | 8.5 |
| *Butyrophilin subfamily 1 member A1** | -8.8-8.5 | 8.1 |
| *Sorting nexin-14* | 9.0 | 8.1 |
| *Phosphatidate phosphatase PPAPDC1A* | 7.8 | 7.9 |
| *Neurotrophin receptor-interacting factor 1* | 7.3 | 7.9 |
| *Endophilin-B1* | 6.5 | 7.6 |
| *Dematin* | 7.3 | 7.5 |
| *Probable ATP-dependent RNA helicase DDX17** | -4.7-8.0 | 7.4 |
| *Sulfatase-modifying factor 1* | 7.6 | 7.4 |
| *Cell surface glycoprotein MUC18* | 6.6 | 7.2 |
| *MBT domain-containing protein 1** | -4.5 | 5.7 |
| *Nuclear factor related to kappa-B-binding protein* | 5.7 | 5.7 |
| *Rho-related GTP-binding protein RhoE** | -5.6-(-4.8) | 5.7 |
| *Macoilin-1* | 5.4 | 5.5 |
| *Receptor-type tyrosine-protein phosphatase F* | 3.6-4.9 | 5.0 |
| *Hemoglobin subunit alpha* | 4.6 | 4.9 |
| *Leukocyte cell-derived chemotaxin-2* | -7.3-(-2.8) | -4.4 |
| *Smoothelin-like protein 2* | -5.4 | -5.3 |
| *Collagen alpha-1(VIII) chain* | -8.4-(5.7) | -5.4 |
| *Complement component C7* | -8.3-(-4.1) | -5.5 |
| *Protein regulator of cytokinesis 1* | -9.6-(-7.1) | -10.7-(-7.3) |
| *Suppressor of cytokine signaling 3* | -8.2-(-4.9) | -7.6 |
| *AT-rich interactive domain-containing protein 5B* | -6.9 | -7.6 |
| *Mannose-binding protein C* | -5.8-(-4.6) | -7.7 |
| *CD9 antigen** | 2.9-7.8 | -7.8 |
| *Feline leukemia virus subgroup C receptor-related protein 1* | -6.6 | -7.9 |
| *Nuclear factor erythroid 2-related factor 2* | -8.0 | -8.0 |
| *H/ACA ribonucleoprotein complex subunit 3** | 3.8-5.5 | -8.2 |
| *Transcription factor COE1* | -7.1 | -8.3 |
| *Mismatch repair endonuclease PMS2** | 7.8-8.1 | -8.5 |
| *Pyruvate kinase PKM* | -8.7 | -8.7 |
| *Peptidyl-prolyl cis-trans isomerase FKBP5* | -6.7-(-2.6) | -9.0 |
| *RNA polymerase II subunit A C-terminal domain phosphatase* | 7.8 | -9.1 |
| *P2X purinoceptor 4* | -9.2-(-2.7) | -9.2 |
| *C-C motif chemokine 2* | -11.1-11.1 | -11.1 |
| ***Differential expression between vaccine groups** | | |
