# Supplementary material for: Vaccine-Induced Protection Against Furunculosis Involves Pre-emptive Priming of Humoral Immunity in Arctic Charr
Source: Front Immunol. 2019 Feb 4;10:120. doi: 10.3389/fimmu.2019.00120 (PMC6369366; doi:10.3389/fimmu.2019.00120)
Supplement: Supplementary file 10 [file Table_10.docx]

**Supplemental Table 10.** Pearson’s correlations between expression of *Asal apoO* and host gene expression of immune mediators.

| ***apoO* vs.** | **Pearson’s *r*** | **p-value** |
| --- | --- | --- |
| *blnk* | -0.296 | 0.00854 |
| *finc* | -0.670 | 2.038e-11 |
| *mx2* | 0.089 | 0.4388 |
| *c7* | -0.113 | 0.3239 |
| *il6rb* | -0.187 | 0.1013 |
| *hsp90b* | -0.097 | 0.3966 |
| *c3* | 0.203 | 0.07459 |
| *hsp90b* | -0.155 | 0.1755 |
| *ladd* | -0.192 | 0.09137 |
