# Supplementary material for: Vaccine-Induced Protection Against Furunculosis Involves Pre-emptive Priming of Humoral Immunity in Arctic Charr
Source: Front Immunol. 2019 Feb 4;10:120. doi: 10.3389/fimmu.2019.00120 (PMC6369366; doi:10.3389/fimmu.2019.00120)

**Supplemental Figure 1.** Intestine of PBS-injected *Asal*-infected controls was highly necrotic, with many pyknotic nuclei and hemorrhage throughout (a). In contrast, vaccinated intestine retained normal architecture (b), and IgM+ lymphocytes were observed in the lamina propria, and migrating through intestinal mucosa (c).

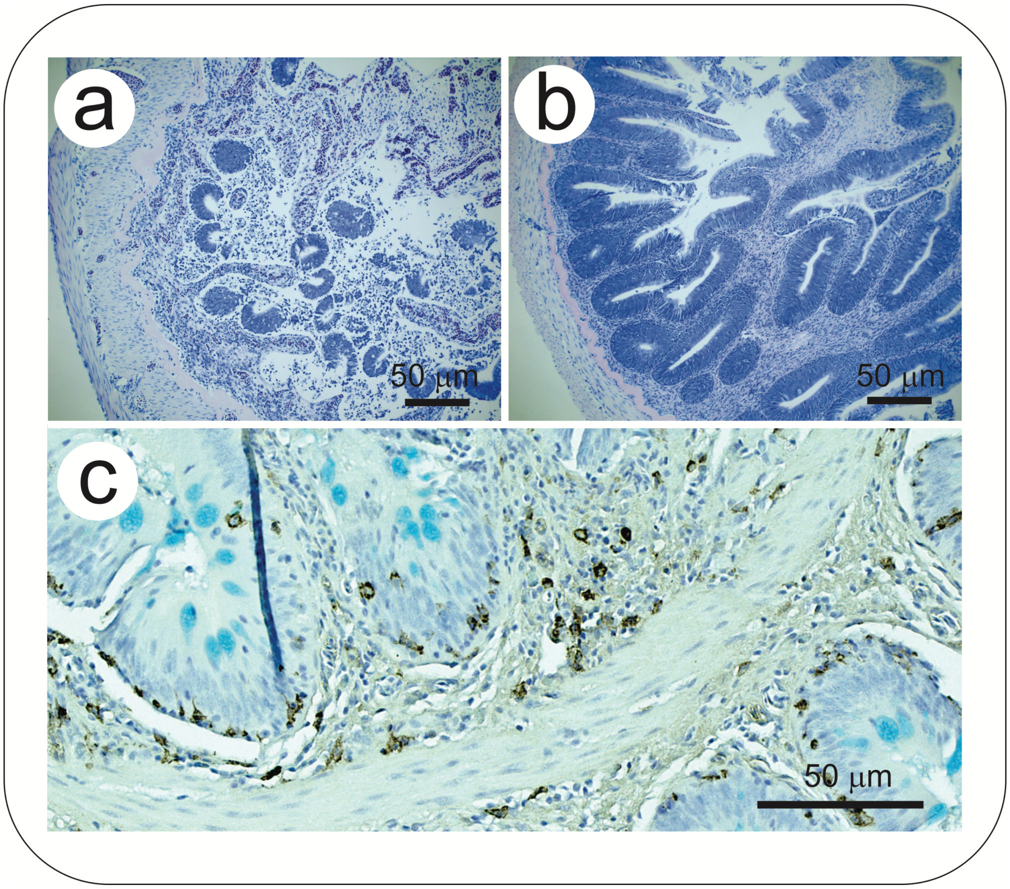

Supplement: Supplementary file 16 [file Image_1.pdf]
