# Supplementary material for: Vaccine-Induced Protection Against Furunculosis Involves Pre-emptive Priming of Humoral Immunity in Arctic Charr
Source: Front Immunol. 2019 Feb 4;10:120. doi: 10.3389/fimmu.2019.00120 (PMC6369366; doi:10.3389/fimmu.2019.00120)

Supplemental Figure 2. Differentially expressed transcripts at 0 dpi/517 ddpv (A), 8 dpi/605 ddpv (B), and 29 dpi/836 ddpv (C), obtained using either *edgeR* or *DESeq2* pipelines.

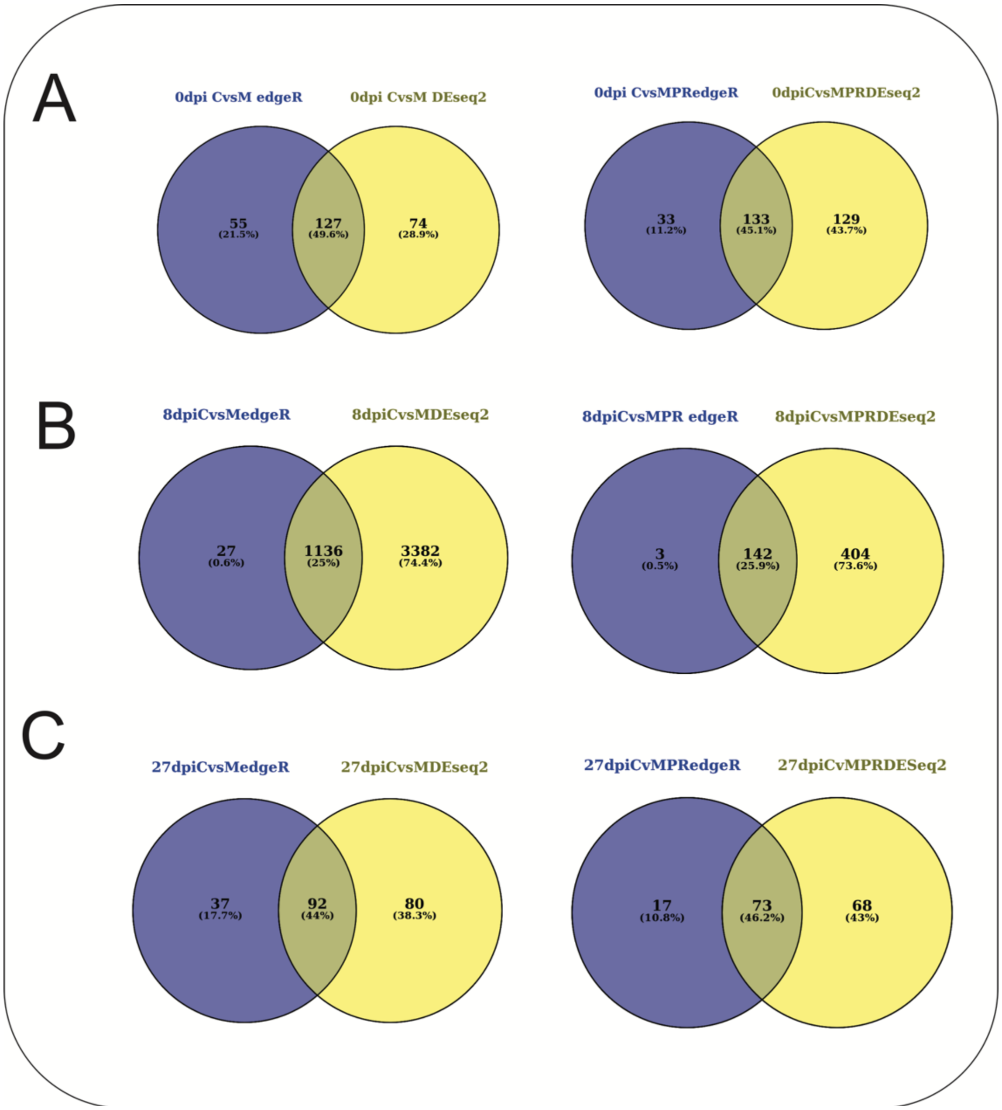

Supplement: Supplementary file 17 [file Image_2.pdf]
