# Supplementary material for: Vaccine-Induced Protection Against Furunculosis Involves Pre-emptive Priming of Humoral Immunity in Arctic Charr
Source: Front Immunol. 2019 Feb 4;10:120. doi: 10.3389/fimmu.2019.00120 (PMC6369366; doi:10.3389/fimmu.2019.00120)

Supplemental Figure 3. Expression profiles of genes involved in the host immune response to *Asal* as identified by RNA-sequencing. Values represent the median log2-fold change with 95% confidence intervals. Significant differences between treatments and over time were identified using a 2-way ANOVA followed by a *post-hoc* Tukey HSD (p<0.05).

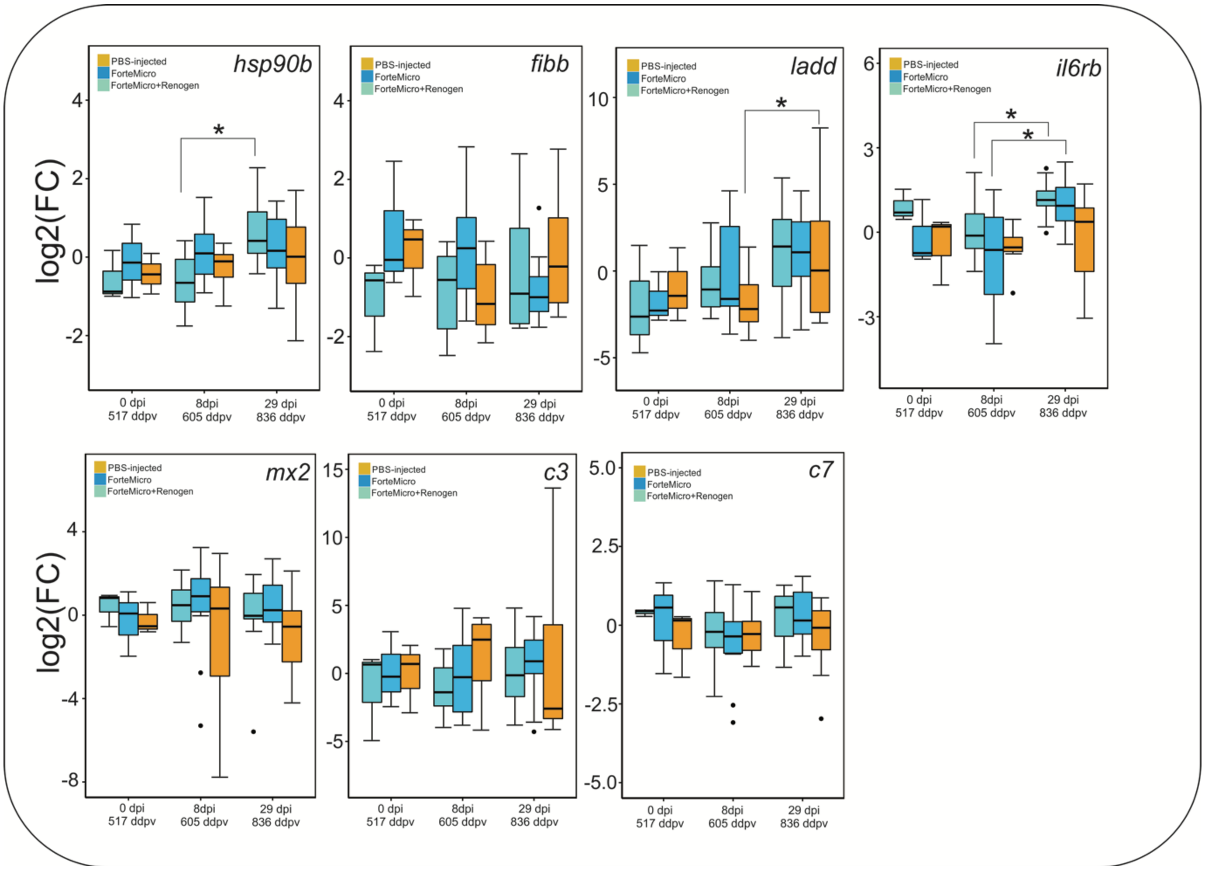

Supplement: Supplementary file 18 [file Image_3.pdf]
